# Cardiovascular Diseases and Cancer: Convergent Proteostasis Networks at the Crossroads of Mechanisms and Therapeutic Opportunities

**DOI:** 10.1002/mco2.70892

**Published:** 2026-08-07

**Authors:** Jacob Eli García Torres, Norbert Frey, Ashraf Yusuf Rangrez

**Affiliations:** ^1^ Department of Cardiology Angiology and Pneumology University Hospital Heidelberg Heidelberg Germany; ^2^ DZHK (German Centre for Cardiovascular Research) Partner Site Heidelberg Mannheim Germany

**Keywords:** autophagy, cancer, cardio‐oncology, cardiotoxicity, CVDs, drug repurposing, ER stress, proteostasis, unfolded protein response, ubiquitin–proteasome system

## Abstract

Cancer and cardiovascular diseases, the primary causes of mortality globally, are increasingly understood as biologically interconnected rather than distinct pathologies. Recent evidence indicates that protein homeostasis (proteostasis) functions as a crucial molecular link connecting tumor progression, therapeutic resistance, cardiac remodeling, and treatment‐related cardiotoxicity. Proteostasis, which encompasses the cellular processes of protein synthesis, folding, quality control, and degradation, dictates tissue adaptation to chronic stress. Notably, the adaptive mechanisms that allow tumor cells to endure proteotoxic stress and resist therapy are often vital for maintaining cardiac structure and function. Thus, tumor control and cardiovascular injury may be divergent outcomes of a common stress–response framework. In this review, we propose proteostasis as a comprehensive framework for understanding the cancer–cardiovascular interface. We analyze how the ubiquitin–proteasome system, autophagy–lysosome pathway, endoplasmic reticulum stress‐induced unfolded protein response signaling, and molecular chaperone networks are differentially reconfigured in cancer and cardiac tissues, influencing tumor survival, therapeutic susceptibility, and cardiovascular dysfunction. Additionally, we explore the translational implications of proteostasis dysregulation, including mechanisms of anticancer therapy‐induced cardiotoxicity, emerging biomarkers, cardioprotective strategies, and opportunities for precision cardio‐oncology. By conceptualizing efficacy and toxicity as interconnected outcomes of shared proteostasis biology, this review establishes a foundation for developing therapies that optimize cancer control while safeguarding cardiovascular health.

## Graphical abstract

The disruption of proteostasis, encompassing the ubiquitin–proteasome system, autophagy, UPR/ER stress, and chaperone function, represents a fundamental mechanism shared by both cancer and cardiovascular diseases. While these pathways frequently facilitate tumor progression, they are crucial for maintaining proteostasis in cardiac tissue. Consequently, cancer treatments that impact proteostasis may induce cardiotoxicity, whereas medications for heart conditions could exhibit anticancer properties.

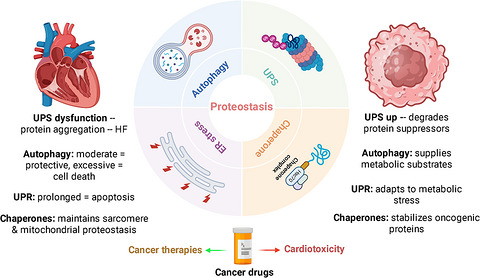

## 1 Introduction

Historically, cardiovascular diseases (CVDs) and cancer have been regarded as distinct clinical and biological entities, each explored within its respective research domain and treated with specific therapies [1, 2, 3]. Traditional cardiovascular research has concentrated on maladaptive responses to hemodynamic stress, metabolic challenges, atherosclerotic inflammation, and ischemia–reperfusion injury. Conversely, cancer research has focused on unchecked cell proliferation, genomic instability, epigenetic modifications, and immune evasion [4, 5]. This historical separation has led to the development of parallel research frameworks and clinical treatment pathways, reinforcing the perception that these disease categories function largely independently [1, 2]. However, over the past two decades, this perspective has been increasingly challenged by converging evidence from epidemiological, clinical, and experimental studies. Comprehensive population‐based research and meta‐analyses have demonstrated strong bidirectional associations between CVDs and cancer, indicating that individuals with pre‐existing CVDs are more likely to develop cancer, while cancer survivors face a significantly elevated long‐term risk of heart failure, arrhythmias, ischemic heart disease, and cerebrovascular events [3, 6, 7, 8, 9]. Notably, these associations persisted even after adjusting for common demographic and lifestyle risk factors such as aging, obesity, smoking, and metabolic issues, suggesting the presence of shared biological mechanisms rather than mere confounding factors [2, 3, 10].

The establishment of cardio‐oncology as a distinct interdisciplinary field has been instrumental in identifying the link between cancer and CVDs. Initially, this field emerged from the need to address the cardiovascular side effects of cancer treatments. Over time, it has broadened to include a comprehensive understanding of the inflammatory, metabolic, thrombotic, and neurohormonal pathways that influence both tumor development and cardiovascular risk [10, 11]. Studies have shown that cardiovascular dysfunction can contribute to tumor growth through circulating factors and systemic inflammatory signals, whereas cancer and its treatments can accelerate cardiovascular aging and disease progression [6, 10]. Clinically, these interactions have significant consequences, as cancer survivors often face increased cardiovascular morbidity and mortality, which can be as high as or higher than their risk of cancer recurrence [7, 8, 9]. Additionally, patients with pre‐existing cardiovascular conditions frequently exhibit reduced tolerance to cancer therapies and poor cancer‐specific outcomes [12, 13].

In addition to shared risk factors, increasing molecular evidence suggests that common cellular stress adaptation mechanisms are central to both cancer and CVDs. A key element among these is protein homeostasis (proteostasis), which involves the coordinated regulation of protein production, folding, transport, and breakdown, and has been identified as a fundamental organizing concept [14, 15, 16, 17]. Proteostasis networks allow cells to endure both internal and external stress; however, their dysfunction can lead to disease progression in various pathological settings. In cancer, the proteostasis system is often hijacked to support tumor growth, manage proteotoxic stress, and aid in therapy resistance [18, 19, 20]. Conversely, the adult heart, primarily composed of terminally differentiated cardiomyocytes with limited regenerative ability, depends on highly precise proteostasis regulation to maintain sarcomeric structure, mitochondrial health, and long‐term contractile performance [21, 22].

This intrinsic imbalance creates a biological conflict at the intersection of cancer and CVDs. Molecular pathways that promote tumor survival and adaptability may simultaneously weaken cardiac resilience, particularly under drug‐induced stress. Numerous modern anticancer treatments, such as proteasome inhibitors (PIs), autophagy regulators, and drugs targeting endoplasmic reticulum (ER) stress responses, directly disrupt the proteostasis network. This disruption explains their dual role in both tumor inhibition and cardiotoxicity [23, 24, 25]. Although these connections are increasingly acknowledged, research has predominantly explored proteostasis mechanisms in cancer and CVDs separately, rather than through a comparative approach that distinguishes shared molecular pathways from context‐specific pathological outcomes.

In this review, a systems‐level perspective is employed to address this gap. By integrating insights from cancer biology, cardiovascular research, and cardio‐oncology, we analyzed how core proteostasis modules, such as the ubiquitin–proteasome system (UPS), autophagy–lysosome pathway, ER stress‐induced unfolded protein response (UPR) signaling, and molecular chaperone networks, are differentially reconfigured to drive divergent but interconnected disease trajectories. Additionally, we investigated how these mechanisms influence therapeutic vulnerability, cardiotoxic liability, and opportunities for drug repurposing and cardioprotective intervention. Through this integrated approach, proteostasis is revealed not only as a shared pathway but also as a conceptual framework for understanding and potentially reconciling the competing demands of effective cancer therapy and long‐term cardiovascular preservation.

## 2 Conceptual Framework

Proteostasis offers a mechanistic framework for understanding the biological convergence of cancer and CVDs. Despite the fundamental differences in their clinical presentations, both conditions are influenced by the ability of cells to maintain proteostasis amid ongoing stress. The adaptive restructuring of proteostasis networks supports tumor survival, progression, and resistance to treatment, whereas disturbances in these networks can undermine cardiac integrity and result in cardiotoxicity. This section positions proteostasis as a systems‐level link between these seemingly unrelated diseases, explores the common molecular foundation of tumor resistance and cardiovascular damage, and presents a unified perspective for integrating disease mechanisms, therapeutic responses, and clinical outcomes across the cancer–cardiovascular spectrum.

### 2.1 Proteostasis as a Systems‐Level Bridge Between Cancer and CVDs

Proteostasis encompasses a complex and interconnected network of cellular mechanisms responsible for the synthesis, folding, transport, posttranslational modification, and degradation of proteins within cells [26]. These processes ensure proteome stability under normal and stressful conditions [14, 27]. Rather than functioning as isolated, linear pathways, proteostasis networks operate as adaptable and responsive systems capable of detecting proteotoxic stress and modulating cellular activities to maintain functional equilibrium [14, 27, 28]. This systemic organizational structure enables proteostasis to act as a molecular link between cancer and CVDs, two distinct conditions marked by persistent and cumulative cellular stress.

In cancer, malignant cells endure persistent proteotoxic stress due to rapid proliferation, genomic instability, aneuploidy, and adverse microenvironmental conditions such as hypoxia, nutrient deprivation, and oxidative stress [29, 30]. To survive and adapt under these conditions, tumor cells actively reprogram proteostasis networks, enhancing their protein quality control capacity while selectively stabilizing oncogenic signaling nodes and managing misfolded protein burdens [18, 20]. This adaptive remodeling imparts significant plasticity, allowing malignant cells to withstand otherwise lethal stress and develop resistance to therapy. Conversely, cardiomyocytes encounter proteostasis stress primarily due to mechanical load, high metabolic demand, ischemia–reperfusion injury, and aging [15, 16, 21]. Unlike cancer cells, adult cardiomyocytes are predominantly postmitotic and irreplaceable, making them particularly susceptible to the disruption of proteome maintenance. Even minor impairments in protein quality control can lead to irreversible sarcomeric disorganization, mitochondrial dysfunction, and progressive contractile failure, ultimately resulting in heart failure [15, 16, 21]. Consequently, the heart depends on stringent proteostasis fidelity rather than adaptive flexibility to maintain long‐term function.

Divergence in the cellular context results in a shared yet fundamentally asymmetric reliance on proteostasis. Cancer cells utilize proteostasis plasticity to facilitate adaptation, survival, and clonal evolution. In contrast, the myocardium relies on the precise and sustained execution of proteostasis processes to maintain its structural and functional integrity. Notably, when the same molecular machinery is pushed beyond its adaptive capacity, it may simultaneously support tumor survival and induce cardiac injury, particularly under the pharmacological stress of contemporary anticancer therapies [18, 23, 24]. This intrinsic tension positions proteostasis as both a shared pathway and a conceptual framework for systematically understanding the biological trade‐offs at the cancer–cardiovascular interface.

### 2.2 Why Tumor Resistance and Cardiotoxicity Share Molecular Roots

In the field of cardio‐oncology, a major issue is the cardiovascular toxicity that cancer treatments can cause, often through mechanisms that are similar to their antitumor actions. This seeming contradiction can be understood by considering tumor resistance and cardiotoxicity as separate results of stress response activation within common proteostasis networks [23, 31, 32]. Stress caused by cytotoxic chemotherapy, targeted therapies, or immunotherapy increases proteotoxic stress, requiring a heavy dependence on adaptive protein quality control pathways for cell survival.

In tumor cells, the activation of proteostasis programs, such as enhanced proteasomal degradation, upregulated autophagy, and the induction of stress‐responsive molecular chaperones, can mitigate drug‐induced damage, stabilize oncogenic signaling, and ultimately foster therapeutic resistance [18, 20]. These adaptations increase the buffering capacity of malignant cells, allowing them to survive under conditions that would otherwise exceed the tolerable proteostasis limits. Conversely, in cardiomyocytes, excessive or prolonged activation of these pathways may overwhelm compensatory reserves, leading to maladaptive remodeling, energetic failure, and cell death [15, 16, 21]. Thus, tumor resistance and cardiotoxicity do not stem from fundamentally distinct molecular targets but rather from the context‐dependent rewiring of the shared stress–response circuitry, which is influenced by cellular identity, regenerative capacity, and baseline proteostasis demand.

This framework elucidates the dual nature of proteostasis‐targeting therapy. PIs exploit the increased reliance of cancer cells on enhanced protein turnover and quality control. However, they may also disrupt the maintenance of the cardiomyocyte proteome, increase oxidative stress, and impair contractile function [15, 18, 33]. Similarly, pharmacological modulation of autophagy or UPR signaling can promote tumor cell death but may destabilize cardiac homeostasis if temporal, dosage, or context‐specific thresholds are exceeded [25, 34, 35]. These findings collectively highlight that cardiotoxicity and tumor resistance are opposing outcomes of the same biological principle: the differential ability of cells to adapt to sustained proteostasis.

### 2.3 Proteostasis As a Unifying Lens for Mechanism–Disease–Therapy Integration

Adopting a proteostasis‐centered framework for examining cancer and CVDs facilitates a transition from mere descriptive cataloging of pathways to genuine mechanistic integration. This approach shifts the focus from labeling signaling pathways as inherently “oncogenic” or “cardioprotective” to analyzing the specific conditions under which proteostasis programs are activated within the cellular context. Such contextualization is crucial for elucidating why identical molecular perturbations, whether genetic, pharmacological, or environmental, can produce vastly different outcomes across various tissues and disease states. Importantly, this framework lays a rational foundation for therapeutic innovation. By pinpointing the nodal points where proteostasis networks diverge between tumor cells and cardiomyocytes, it is feasible to design context‐sensitive interventions that exploit cancer‐specific dependencies while maintaining cardiac proteome integrity. This rationale underpins strategies such as the temporal or dose modulation of proteostasis‐targeting agents, formulation of rational drug combinations, and implementation of cardioprotective cotherapies that mitigate stress–response overload without diminishing anticancer efficacy. Furthermore, disruptions in proteostasis signaling may represent an underutilized source of biomarkers that can facilitate early cardiotoxicity risk stratification, dynamic treatment monitoring, and prediction of therapeutic responses.

When considered collectively, proteostasis is revealed not just as a common molecular characteristic of cancer and CVDs, but as a systems‐level organizing principle that integrates disease mechanisms, therapeutic susceptibilities, and clinical results [26]. This conceptual basis provides a unified framework for the subsequent sections, which explore specific proteostasis modules in depth and analyze how their context‐specific reconfiguration leads to distinct yet interrelated pathways at the intersection of cancer and CVDs.

## 3 Core Proteostasis Pathways at the Cancer–CVDs Interface

The biological interaction between cancer and CVDs is governed by a limited number of highly interconnected proteostasis pathways responsible for protein quality control, stress adaptation, and cell fate determination [26]. The UPS, autophagy–lysosome pathway, and UPR are the primary mechanisms that maintain proteome integrity under both physiological and pathological conditions. Although these pathways are activated in tumor cells and cardiomyocytes in response to proteotoxic stress, their functional outcomes are significantly context‐dependent. In cancer, proteostasis remodeling often facilitates survival, adaptation, and resistance to therapy. Conversely, prolonged or dysregulated activation in the heart typically leads to maladaptive remodeling and functional deterioration. This section explores how these fundamental proteostasis modules influence the shared yet distinct biology of tumor progression and cardiovascular injury at the cancer–cardiovascular interface.

### 3.1 UPS: Proteotoxic Stress, Inflammation, and Remodeling

The UPS is the primary intracellular mechanism for the selective degradation of proteins, which is essential for maintaining proteome stability under both normal and adverse conditions [36, 37, 38, 39]. This system ensures substrate specificity and precise timing of protein turnover through the coordinated actions of E1 ubiquitin‐activating enzymes, E2 ubiquitin‐conjugating enzymes, and E3 ubiquitin ligases. These enzymes tag damaged, misfolded, or short‐lived regulatory proteins for degradation by the 26S proteasome complex. Beyond its conventional role in quality control, the UPS significantly impacts signal transduction, cell cycle progression, inflammatory signaling, and stress adaptation, positioning it at a critical intersection between cancer biology and cardiovascular stability [40, 41, 42].

In the context of cancer, the increased reliance on proteasomal degradation arises from the necessity to manage elevated protein synthesis, genomic instability, and enhanced oncogenic signaling [29, 30, 43]. Cancer cells exploit the UPS to eliminate misfolded proteins resulting from aneuploidy and rapid growth while stabilizing oncogenic drivers through selective ubiquitination or deubiquitination [18, 44, 45]. This proteostasis modulation facilitates continuous growth, survival under proteotoxic stress, and resistance to treatment. Conversely, disruption of UPS function results in toxic protein accumulation, ER stress, and apoptotic signaling, a vulnerability that has been therapeutically targeted, particularly through proteasome inhibition in multiple myeloma and related hematological malignancies [20, 46, 47].

In the cardiovascular system, the UPS is equally vital but serves distinct roles. Cardiomyocytes rely on tightly regulated ubiquitin‐mediated degradation to remove damaged sarcomeric proteins, regulate calcium‐handling machinery, and maintain mitochondrial quality control in response to persistent mechanical and metabolic stress [15, 48, 49]. Disruption of UPS activity has been associated with pathological cardiac hypertrophy, ischemia–reperfusion injury, and heart failure, where both insufficient proteasomal degradation and excessive proteolysis can destabilize contractile structures, impair excitation–contraction coupling, and initiate inflammatory remodeling of the heart [50, 51, 52, 53, 54]. These findings underscore the narrow therapeutic window within which UPS activity must be maintained to preserve the cardiac function.

A key characteristic of UPS biology at the intersection of cancer and CVDs is the context‐specific nature of ubiquitination. The effects of ubiquitin attachment are influenced not only by the specificity of the substrate but also by the topology of the linkage between ubiquitin molecules. Typically, K48‐linked polyubiquitination directs proteins toward proteasomal degradation, whereas K63‐linked and other nonstandard ubiquitin chains mainly influence protein interactions and signaling pathways [55, 56]. This difference is illustrated by the regulation of protein kinase B (AKT), which is crucial in both cancerous and heart‐protective signaling. K48‐linked ubiquitination of AKT, driven by tumor suppressors such as phosphatase and tensin homolog (PTEN), aids in its breakdown and limits tumor growth [57, 58, 59, 60]. Conversely, K63‐linked ubiquitination of AKT boosts kinase activation and downstream signaling without causing degradation, thus encouraging tumor development [61, 62].

**FIGURE 1:**
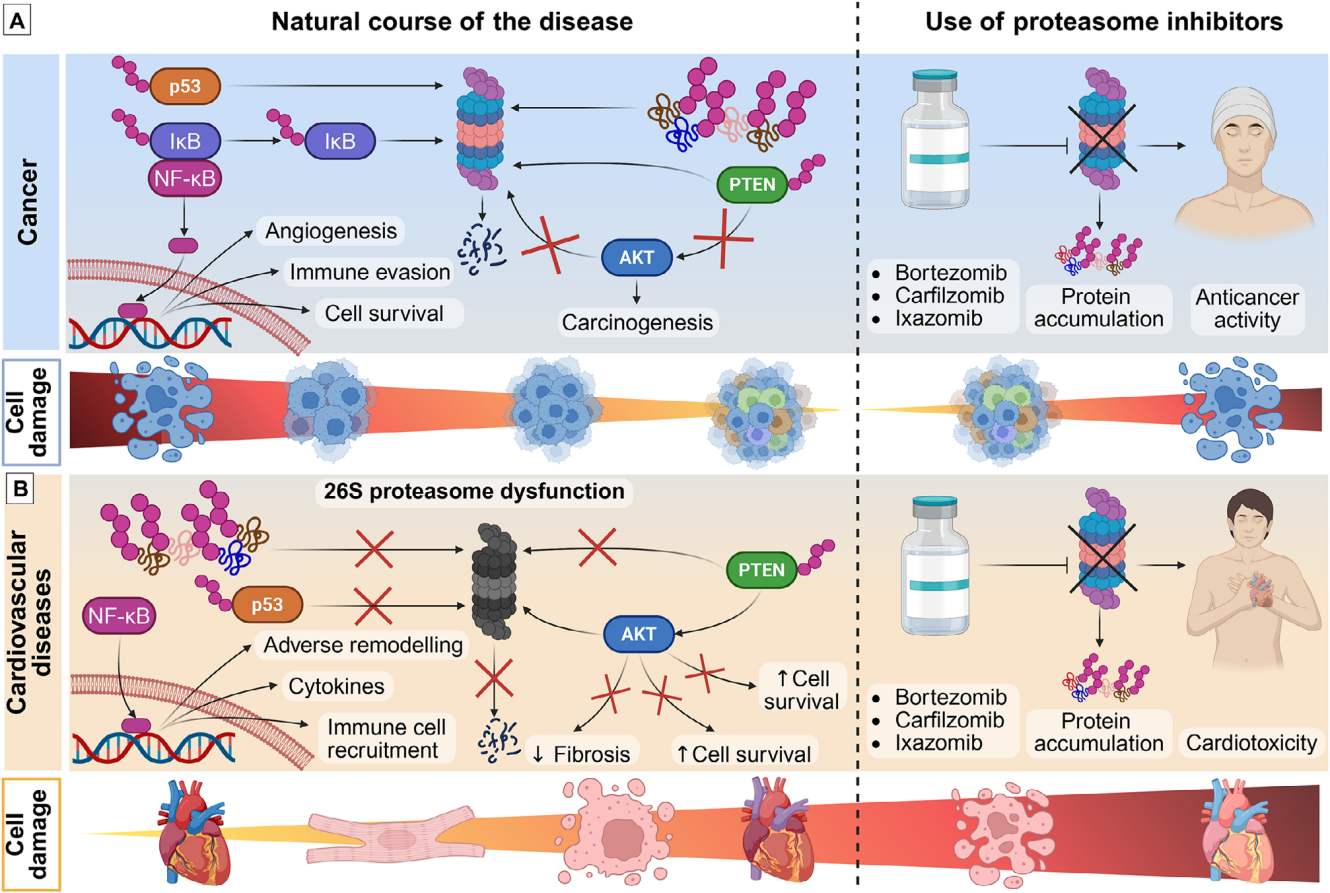
Intersection of the ubiquitin–proteasome system (UPS) in cancer and cardiovascular diseases (CVDs). (A) The ubiquitin–proteasome pathway facilitates the breakdown of key tumor suppressor proteins and inhibitors of specific transcription factors, thereby enhancing cell survival and tumorigenesis. By causing the accumulation of damaged proteins that are not degraded, proteasome inhibitors reduce cancer cell viability by inducing proteotoxicity. (B) The 26S proteasome is essential for maintaining cellular function in the cardiovascular system. Malfunctioning of this process can result in the accumulation of misfolded and unfolded proteins, which may lead to CVDs. Proteasome inhibitors can easily exceed the limited proteotoxic stress tolerance of cardiomyocytes, potentially causing long‐term cardiotoxicity. *Abbreviations*: protein kinase B (AKT); phosphatase and tensin homolog (PTEN); inhibitor of κB (IκB); and nuclear factor‐κB (NF‐κB). Created with BioRender.com.

Interestingly, the functional equilibrium of this pathway varies significantly from the cardiac viewpoint. In models of cardiac ischemia and stress adaptation, reducing or losing PTEN activity enhances AKT signaling, improves heart function, decreases fibrosis, and limits cardiomyocyte apoptosis [63, 64]. This contrast creates a therapeutic dilemma in which heart‐protective treatments might simultaneously increase cancer risk, underscoring the challenge of targeting shared UPS‐regulated nodes across different tissues (Figure 1).

Beyond signaling for growth and survival, the UPS plays a crucial role in regulating inflammation in both cancer and CVDs. The degradation of inhibitor of κB (IκB) by the proteasome is necessary for activating nuclear factor‐κB (NF‐κB), a key transcriptional regulator of inflammatory and immune responses [65, 66, 67]. In tumors, continuous NF‐κB activation aids immune evasion, angiogenesis, and resistance to cell death, whereas in the heart, it influences immune cell recruitment, cytokine production, and adverse remodeling after injury [68, 69, 70]. Chronic disruption of UPS‐mediated inflammatory signaling can exacerbate harmful inflammation in failing hearts while promoting tumor growth and resistance to therapy.

Overall, the UPS demonstrates how a single proteostasis pathway can lead to contrasting biological outcomes based on the cellular context, stress levels, and timing. In cancer, increased UPS flexibility supports survival and adaptation under proteotoxic stress, whereas in heart cells, ongoing UPS dysfunction can lead to irreversible structural damage and functional decline. This dual nature positions the UPS as both an attractive therapeutic target and a significant risk factor at the cancer–cardiovascular interface, paving the way for further discussions on autophagy–lysosome signaling, ER stress responses, and molecular chaperone networks.

### 3.2 Autophagy–Lysosome Pathway: Survival Versus Maladaptation

Autophagy is an evolutionarily conserved, lysosome‐dependent degradation pathway that facilitates the turnover of long‐lived proteins, damaged organelles, and protein aggregates, and serves as a central component of cellular proteostasis [71, 72, 73, 74]. It supports metabolic flexibility and cellular survival by dynamically responding to nutrient availability, energy demands, and stress intensity. Nevertheless, the functional outcomes of autophagic activation are highly dependent on context and duration, positioning autophagy as a crucial determinant of divergent outcomes in cancer and CVDs.

**FIGURE 2:**
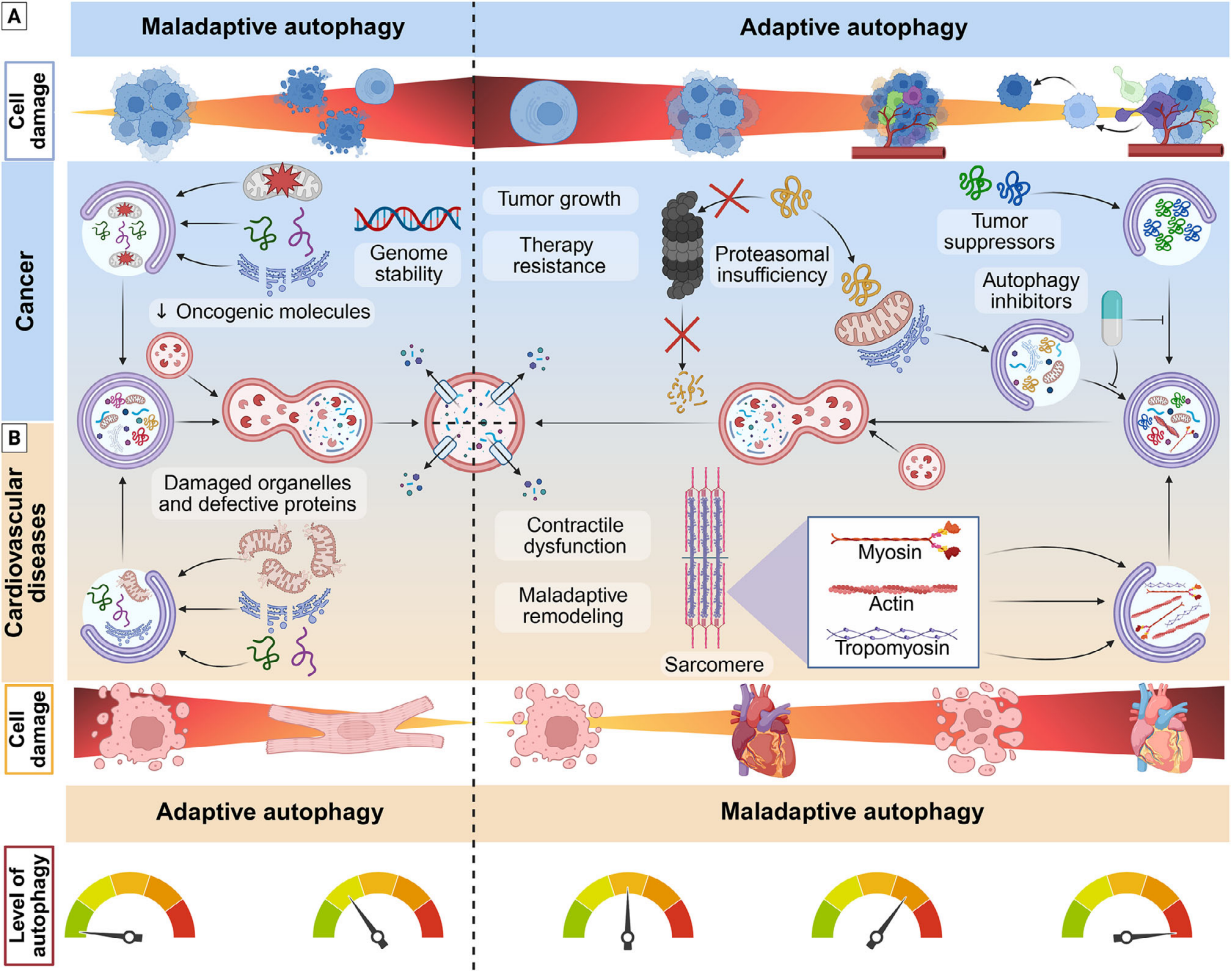
Intersection of autophagy in cancer and cardiovascular diseases (CVDs). (A) In the initial phases of cancer, autophagy prevents tumor formation by reducing genomic instability. As cancer progresses, increased and sustained autophagy meets the energy needs of cancer cells and mitigates proteotoxic stress, particularly when proteasome function is compromised. (B) In cardiac tissue, the range of beneficial autophagy is much narrower; both insufficient and excessive autophagy levels are harmful to cardiomyocytes, contributing to the pathophysiology of CVDs, particularly with chronic persistence. This imbalance can lead to the breakdown of vital contractile proteins, causing adverse cardiac effects during anticancer treatment. Created with BioRender.com.

In cancer, autophagy plays a dual and stage‐dependent role. During early tumorigenesis, basal autophagic flux suppresses malignant transformation by limiting oxidative stress, preserving mitochondrial integrity, and maintaining genomic stability [34, 75, 76]. Conversely, in established tumors, autophagy is often upregulated and repurposed to support survival under hypoxic, nutrient‐deprived, and therapeutic pressure [77, 78, 79, 80]. By recycling intracellular macromolecules, autophagy fuels anabolic metabolism, sustains mitochondrial function, and mitigates proteotoxic stress, thereby promoting tumor progression and resistance to chemotherapy, targeted therapies, and radiation [34, 77, 78]. This adaptive reliance positions autophagy as a central contributor to tumor fitness and therapeutic evasion (Figure 2).

In the cardiovascular system, autophagy plays an essential housekeeping role, particularly in cardiomyocytes exposed to continuous mechanical load and high metabolic demand [21, 22, 25, 81, 82]. Basal autophagic flux preserves sarcomeric organization, regulates mitochondrial quality control through mitophagy, and prevents the accumulation of toxic protein aggregates [83]. Genetic or pharmacological disruption of autophagy is causally linked to pathological cardiac hypertrophy, ischemia–reperfusion injury, and heart failure [22, 81, 84]. Notably, both insufficient autophagic activity and excessive, uncontrolled autophagic flux can destabilize cardiomyocyte homeostasis, highlighting the necessity for precise temporal and quantitative regulation of autophagy [22, 25, 84] (Figure 2).

At the intersection of cancer and CVDs, the findings indicate that autophagy has been found to possess limited adaptive capacity. A moderate increase in autophagy can enhance stress tolerance in both tumor cells and cardiomyocytes; however, prolonged or uncontrolled activation leads to distinct pathological effects on both cell types. In cancer cells, persistent autophagy enhances cellular resilience and resistance to therapy, whereas in cardiomyocytes, extended autophagic stress exacerbates energy depletion, promotes maladaptive remodeling, and induces contractile dysfunction [85, 86, 87]. This contrast illustrates how identical autophagic processes can support survival in one tissue type while impeding it in another tissue type.

Autophagy is intricately linked to other proteostasis pathways, such as the UPS and UPR. Insufficient proteasomal function often results in compensatory autophagic activation, whereas ER stress‐induced UPR signaling can initiate autophagic activity as an adaptive mechanism to manage the accumulation of misfolded proteins [32, 88]. In the context of anticancer treatments, the simultaneous disruption of these pathways may elevate proteotoxic stress beyond compensatory levels, resulting in both tumor cell death and unintended cardiotoxic effects [31].

From a therapeutic standpoint, the modulation of autophagy presents a complex challenge. Pharmacological inhibition of autophagy can increase tumor susceptibility to chemotherapy and targeted treatments; however, it may reduce cardiac stress tolerance when used systemically [89, 90, 91]. Conversely, autophagy activation has been explored as a cardioprotective strategy in ischemic and metabolic heart conditions, raising concerns about its potential tumor‐promoting effects in patients with active or dormant cancers [92, 93, 94]. These contrasting outcomes highlight the necessity of designing context‐sensitive therapies with precise dosing and timing when targeting autophagy at the intersection of cancer and cardiovascular health.

In summary, autophagy exemplifies the proteostasis paradox central to cardio‐oncology: a pathway crucial for cellular adaptation that can become detrimental when persistently activated or manipulated therapeutically. Understanding the molecular and contextual factors that dictate the transition from protective recycling to harmful self‐digestion is essential for developing autophagy‐targeted strategies that are both safe and effective for clinical application in humans.

### 3.3 UPR and ER Stress: Adaptive Versus Terminal Signaling

The ER is the primary cellular site for protein folding, lipid synthesis, and calcium homeostasis. Disruptions in ER function, collectively termed ER stress, result in the accumulation of improperly folded or unfolded proteins, thereby activating the UPR, a conserved signaling network designed to restore proteostasis [95, 96, 97, 98]. UPR is regulated by three stress sensors located in the ER: inositol‐requiring enzyme 1 (IRE1), protein kinase RNA‐like ER kinase (PERK), and activating transcription factor 6 (ATF6). Each sensor initiates distinct, yet interconnected, transcriptional and translational responses [99, 100, 101]. Under conditions of mild or transient ER stress, UPR signaling operates adaptively to restore homeostasis. PERK‐induced phosphorylation of eukaryotic initiation factor 2α (eIF2α) temporarily reduces global protein synthesis, thereby alleviating the proteotoxic load while permitting the selective translation of stress‐responsive mRNAs [102, 103]. Concurrently, IRE1‐mediated splicing of X‐box binding protein 1 (XBP1) and ATF6 activation enhance ER folding capacity, stimulate lipid synthesis, and activate ER‐associated degradation and autophagy pathways to facilitate the clearance of misfolded proteins [104, 105, 106, 107]. Collectively, these processes restore ER homeostasis and support cell survival. However, if ER stress becomes severe, prolonged, or recurrent, UPR signaling may transition from a protective to a terminal phase [35]. Persistent activation of PERK–eIF2α signaling can inhibit the protein synthesis required for cell maintenance, while prolonged IRE1 and ATF6 activity may initiate inflammatory, mitochondrial, and apoptotic pathways [98, 102, 108, 109, 110]. This transition from adaptive to terminal response is a defining feature of UPR biology and accounts for its diverse roles in various tissues and disease contexts.

**FIGURE 3:**
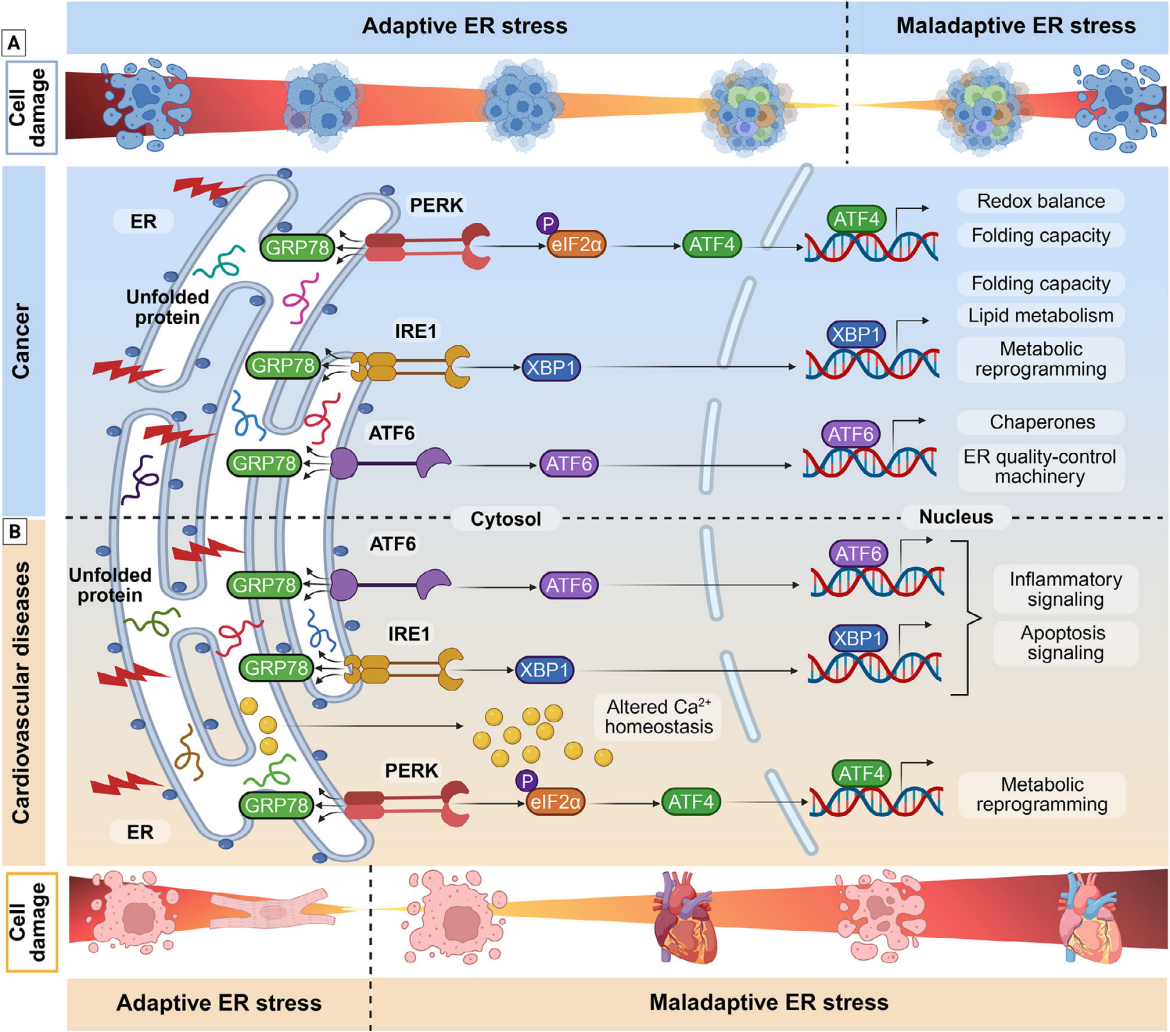
Role of endoplasmic reticulum (ER) stress in cancer and cardiovascular diseases (CVDs). In both cancer and CVDs, the ER chaperone GRP78 separates from the ER stress sensors PERK, IRE1, and ATF6, triggering the UPR to reestablish ER balance via the PERK–eIF2a–ATF4, IRE1–XBP1, and ATF6 pathways. (A) In cancer, this activation enhances chaperone expression, reprograms metabolism, and increases the protein folding capacity. (B) In contrast, cardiomyocytes have a significantly narrower adaptive range than cancer cells. This limited adaptability renders them more vulnerable to damage from inflammation, disrupted calcium balance, and subsequent apoptosis, particularly in response to anticancer therapy. *Abbreviations*: inositol‐requiring enzyme 1 (IRE1); protein kinase RNA‐like ER kinase (PERK); activating transcription factor 6 (ATF6); eukaryotic initiation factor 2α (eIF2α); X‐box binding protein 1 (XBP1); and endoplasmic reticulum (ER). Created with BioRender.com.

In cancer, UPR pathways are frequently co‐opted to facilitate the survival of malignant cells under sustained stress conditions, such as hypoxia, nutrient deprivation, oncogene‐induced protein overload, and therapeutic stress [111, 112, 113, 114]. Activation of the IRE1‐XBP1 axis enhances the ER protein‐folding capacity, lipid metabolism, and metabolic reprogramming, while PERK‐mediated translational regulation assists in maintaining redox balance and mitigating proteotoxic stress [115, 116, 117]. Furthermore, ATF6 augments the production of molecular chaperones and the ER quality control system [118, 119]. Together, these adaptations enable tumor cells to withstand persistent ER stress and develop resistance to chemotherapy, targeted therapy, and immunotherapy [120, 121, 122, 123] (Figure 3A).

In the cardiovascular system, UPR signaling is meticulously regulated in terms of its intensity and duration. Temporary UPR activation can confer cardioprotection during acute stress events, such as ischemia–reperfusion injury, by reducing protein misfolding, maintaining calcium homeostasis, and preserving ER integrity [124, 125, 126, 127]. To the contrary, prolonged or unregulated UPR activation in cardiac myocytes is closely associated with pathological remodeling, impaired contractility, and progression to heart failure [128, 129, 130]. Chronic PERK signaling can disrupt the synthesis of proteins essential for sarcomere maintenance, whereas sustained IRE1 or ATF6 activation may enhance inflammatory and apoptotic signaling, resulting in irreversible cardiac damage [102, 109, 128] (Figure 3B).

At the cancer–cardiovascular interface, the UPR exemplifies a stress‐threshold phenomenon. Tumor cells exploit prolonged UPR activation to enhance their adaptability and survival, whereas cardiac myocytes, due to their limited regenerative capacity and stringent proteostasis requirements, are particularly vulnerable to extended ER stress [128, 129, 131, 132]. Consequently, anticancer therapies that exacerbate ER stress or directly target UPR components can be effective against tumors but may compromise cardiac proteostasis, leading to therapy‐induced cardiotoxicity [16, 97].

In summary, the UPR functions as a molecular switch that integrates the magnitude and duration of stress with the cellular context to dictate the cell fate. Understanding the transition from adaptive UPR signaling to terminal outcomes across various tissues is essential for devising strategies that selectively destabilize tumor proteostasis while preserving the cardiac function.

### 3.4 Molecular Chaperone Networks as Integrators of Proteostasis

Molecular chaperones are essential components of a highly conserved and hierarchically organized proteostasis network. They are crucial for guiding the folding of nascent polypeptides, refolding proteins denatured by stress, and managing the sorting of damaged or aggregation‐prone proteins for either refolding or degradation [133, 134]. Beyond merely assisting in folding, chaperones such as the heat shock protein 70 (HSP70) and heat shock protein 90 (HSP90) families serve as key decision‐makers within the proteostasis network [135, 136]. These molecular chaperones process stress signals and determine the fate of proteins based on the context, dynamically interacting with the UPS, autophagy–lysosome pathway, and UPR [98, 137, 138].

**FIGURE 4:**
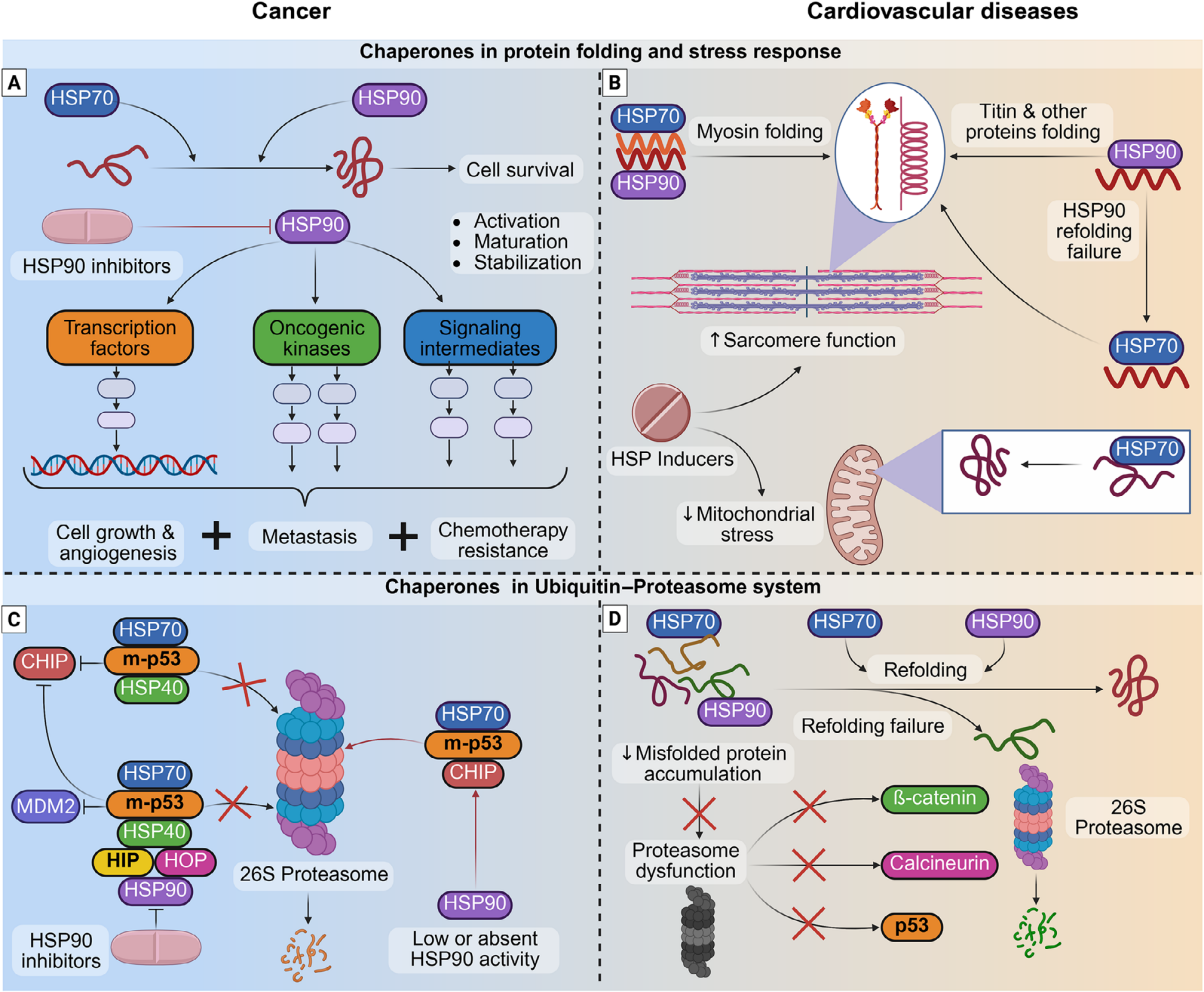
Function of chaperones in maintaining proteostasis in cancer and cardiovascular diseases (CVDs). (A) In cancer, chaperones such as HSP70 and HSP90 aid tumor development by enabling the activation, maturation, and stabilization of various oncogenic proteins. Combining HSP inhibitors with other treatments may disrupt oncogenic pathways while potentially safeguarding cardiac function in some cases. (B) In heart muscle cells, chaperone‐assisted refolding of crucial contractile and mitochondrial proteins enhances contractile performance and cellular balance. (C) Chaperone complexes stabilize mutant p53, preventing its ubiquitination and subsequent degradation by the proteasome, thus encouraging tumor growth. When HSP90 activity is reduced or absent, CHIP forms complexes with HSP70 and mutant p53, leading to the polyubiquitination of mutant p53 for proteasomal degradation. (D) In CVDs, HSP70 and HSP90 contribute to the maintenance of proteasome function and reduction of the accumulation of factors that promote hypertrophy and apoptosis. *Abbreviations*: heat shock protein (HSP); HSP70/HSP90 organizing protein (HOP); carboxyl terminus of HSC70‐interacting protein (CHIP), mouse double minute 2 homolog (MDM2); HSP70‐interacting protein (HIP); and mutant p53 (m‐p53). Created with BioRender.com.

In cancer, chaperone networks are frequently overexpressed, restructured, and exploited to stabilize oncogenic client proteins that are typically targeted for degradation. HSP90 maintains the structural integrity of a diverse array of oncogenic kinases, transcription factors, and signaling intermediates, thereby mitigating the proteotoxic effects of mutations, aneuploidy, and oncogene‐induced overexpression [139] (Figure 4A). Simultaneously, members of the HSP70 family alleviate proteotoxic stress associated with rapid cell proliferation and adverse microenvironmental conditions, thus supporting tumor cell survival and resistance to treatment [140, 141]. A notable example of chaperone‐facilitated oncogenic stabilization is the mutant p53. While wild‐type p53 is stringently regulated through ubiquitination and proteasomal degradation, mutant p53 proteins are selectively shielded by HSP90–HSP70 chaperone complexes, preventing their degradation and enabling gain‐of‐function activities that drive tumor progression, invasion, and resistance to therapy [142, 143, 144]. This targeted preservation of abnormal proteins demonstrates how chaperone networks provide malignant cells with enhanced signaling capabilities under persistent proteotoxic stress [145, 146] (Figure 4C).

In the cardiovascular system, molecular chaperones are essential for protection and maintenance of homeostasis. Cardiomyocytes rely on both inducible and constitutive chaperone functions to maintain protein quality, organize sarcomeres, protect mitochondrial health, and ensure proper functioning of calcium‐handling systems during mechanical, metabolic, and oxidative stress [147, 148, 149, 150]. Proteins such as HSP70, HSP90, and small HSPs, including αB‐crystallin, are crucial for reducing ischemia–reperfusion damage, mitigating remodeling due to pressure overload, and preventing the aggregation of misfolded contractile proteins [151, 152]. Unlike cancer cells, cardiomyocytes do not utilize chaperone networks to support pathological growth; rather, insufficient chaperone induction or dysfunction accelerates the accumulation of proteotoxic substances and mitochondrial issues, leading to worsening cardiac failure (Figure 4B,D).

The diverse roles of molecular chaperones in different tissues underscore their function as integrative triage centers within the proteostasis network. Chaperones actively determine whether damaged proteins should be refolded, isolated, or degraded by interacting with cochaperones, E3 ubiquitin ligases, deubiquitinases, and autophagy adaptors [133, 137, 138]. In cancerous tissues, this triage favors the stabilization and retention of oncogenic proteins, whereas in cardiomyocytes, it focuses on maintaining the structural and metabolic integrity. Consequently, therapeutic modifications of chaperone activity yield significantly different outcomes based on the cellular context, stress levels, and regenerative capacities. These differences are particularly important when considering chaperone‐targeting therapy. Pharmacological inhibition of HSP90 simultaneously disrupts multiple oncogenic signaling pathways and has demonstrated antitumor efficacy in both preclinical models and clinical trials [153, 154]. However, widespread suppression of chaperone function can disrupt cardiac proteostasis, increase ER stress, and render the heart more susceptible to additional damage, highlighting the cardiotoxic risks of broadly targeting chaperone networks [155]. This dual nature emphasizes the necessity of context‐sensitive intervention strategies, such as selective targeting, timing adjustments, and cardioprotective cotherapies.

Overall, molecular chaperones serve as integrators of proteostasis, enhancing or buffering stress responses according to cellular identity and needs. Their central role in the proteostasis network makes them attractive yet inherently risky therapeutic targets, underscoring the broader principle that effective modulation of proteostasis in cancer must be carefully balanced against the unique vulnerabilities of the cardiovascular system.

## 4 Shared Versus Disease‐Specific Outcomes of Proteostasis Dysregulation

A fundamental paradox at the cancer–cardiovascular interface is that identical proteostasis pathways can simultaneously promote survival in one tissue while inducing dysfunction in the other. This contradiction highlights significant differences in how tumor cells and cardiomyocytes engage with, tolerate, and resolve proteotoxic stress. Cancer cells frequently exploit the plasticity of proteostasis to sustain growth, adaptation, and resistance to therapy, whereas cardiomyocytes rely on precise maintenance of proteostasis to preserve structural integrity and long‐term function. Therefore, the effects of proteostasis dysregulation are influenced not only by pathway activation but also by cellular context, adaptive capacity, and stress burden. This section analyzes how shared proteostasis machinery leads to divergent disease phenotypes through context‐dependent signaling rewiring and variations in stress tolerance thresholds and discusses the implications of these mechanisms for therapeutic targeting in cardio‐oncology.

### 4.1 Common Molecular Machinery, Divergent Cellular Consequences

The proteostasis network, encompassing the UPS, autophagy–lysosome pathway, UPR, and molecular chaperone systems, represents a highly conserved mechanism across mammalian cells. It operates as an interconnected set of modules that detect, buffer, and clear stress to maintain proteome integrity [14, 27]. Notably, the biological outcomes of activating these pathways vary significantly between cancer cells and cardiomyocytes. This variation arises from fundamental differences in cellular identity, growth potential, inherent proteotoxic stress, energy constraints, and regenerative capacity. Such divergence provides a mechanistic explanation for a common clinical observation in cardio‐oncology: identical molecular changes can be advantageous in tumor treatment but detrimental to cardiac health.

Cancer cells endure chronic proteotoxic stress due to rapid proliferation, genomic instability, aneuploidy, and intense oncogenic signaling. These factors elevate the burden of misfolded proteins, destabilize protein complexes, and increase reliance on adaptive proteostasis capacity. Consequently, cancer cell survival is closely linked to their ability to reconfigure proteostasis networks under persistent stress, facilitating continued growth and therapy evasion. Conversely, cardiomyocytes are highly susceptible to proteostasis disruptions. They are long‐lived, mechanically active, and possess high metabolic demands, with limited renewal capacity over a human lifespan. Their proteome is dominated by large, highly organized macromolecular structures, particularly the sarcomere, and organelle systems that must function continuously to maintain excitation–contraction coupling. Therefore, cardiomyocytes depend more on proteostasis fidelity than flexibility, necessitating precise and sustained execution of folding, triage, and degradation processes to prevent cumulative damage. Even minor disruptions in turnover or folding can destabilize sarcomeric structure, impair calcium handling, compromise mitochondrial quality control, and initiate remodeling programs that become self‐perpetuating.

This asymmetry between cancer and cardiac cells elucidates why interventions enhancing proteostasis can yield opposite effects in different tissues. In tumors, increased engagement of stress responses can be adaptive, mitigating therapy‐induced proteotoxicity and preserving oncogenic signaling. In cardiomyocytes, however, the same sustained engagement often signifies a system operating at or beyond its compensatory limits, where ongoing stress signaling becomes maladaptive and leads to dysfunction. The key implication is that plasticity tends to benefit malignancy, while fidelity safeguards cardiac function, positioning proteostasis at the center of a mechanistic trade‐off that must be carefully considered when developing therapies, dosing schedules, and cardioprotective strategies at the intersection of cancer and CVDs.

### 4.2 Context‐Dependent Rewiring of Proteostasis Signaling

The context‐dependent modification of signaling pathways is a crucial factor influencing disease‐specific outcomes at the intersection of cancer and CVDs. In cancer, proteostasis mechanisms are often altered to promote adaptation and survival, primarily through the persistent activation of stress response pathways that are typically intolerable for most normal tissues. Sustained engagement of autophagy or UPR signaling allows tumor cells to endure conditions such as hypoxia, nutrient deprivation, proteotoxic stress induced by oncogenes, and therapeutic interventions, while simultaneously inhibiting apoptotic processes that would otherwise compromise cell viability.

Conversely, these signaling patterns yield markedly different outcomes in cardiomyocytes. Prolonged autophagy may transition from a protective recycling role to harmful self‐digestion and energy depletion, whereas continuous UPR activation can shift from alleviating stress to inducing inflammation, mitochondrial dysfunction, and proapoptotic signaling pathways. Unlike tumor cells, cardiomyocytes lack the ability to compensate for damaged proteins through proliferation, rendering them particularly susceptible to gradual proteostasis imbalance over time. This highlights a fundamental principle in cardio‐oncology proteostasis: the duration, intensity, and cellular context of stress–response activation, rather than the specific pathways themselves, determines whether proteostasis signaling supports survival or results in irreversible dysfunction.

### 4.3 Proteostasis Thresholds and Stress Tolerance Windows

The concept of proteostasis stress tolerance thresholds offers a valuable framework for elucidating why conserved proteostasis mechanisms yield diverse cellular outcomes in cancer and CVDs. Tumor cells often augment their proteostasis capabilities by concurrently upregulating molecular chaperones, enhancing proteasome activity, and promoting autophagic degradation. This adaptation raises the threshold at which proteotoxic stress becomes lethal, enabling cancer cells to endure chronic protein overload induced by oncogenes and adverse microenvironmental conditions. This adaptive enhancement is crucial for both innate and acquired resistance to cancer therapy.

On the other hand, cardiomyocytes function within a relatively narrow proteostasis range, optimized to maintain structural integrity over the long term rather than immediate adaptability. As terminally differentiated cells with mechanical functions and limited regenerative capacity, cardiomyocytes depend on stringent quality control systems to preserve sarcomeric integrity, mitochondrial function, and excitation–contraction coupling over extended periods. Stressors, whether therapeutic or pathological, that exceed this limited proteostasis range can precipitate rapid functional decline through maladaptive remodeling, energy failure, and progressive contractile dysfunction, even in the absence of immediate cell death.

This imbalance creates a fundamental cardio‐oncologic trade‐off: treatments that exceed the proteostasis capacity of tumors, such as PIs, prolonged ER stress induction, or disruption of chaperone buffering, may also destabilize cardiac homeostasis when administered either systemically or chronically. Therefore, it is crucial to delineate tissue‐specific stress tolerance limits and develop strategies to selectively target tumor proteostasis dependencies while preserving myocardial stability to ensure safe therapeutic interventions at the intersection of cancer and CVDs.

### 4.4 Implications For Cardio‐Oncology and Therapeutic Targeting

Mechanistic differences in proteostasis have substantial implications for cardio‐oncology. Therapies targeting proteostasis are specifically designed to exploit the dependence of tumors on stress‐buffering mechanisms, chaperone support, proteasomal activity, and adaptive autophagy/UPR engagement. However, these therapies are often applied in physiological contexts where cardiac muscle has a limited capacity to endure prolonged proteostasis disruption. Consequently, cardiotoxicity frequently emerges not only as an unintended side effect but also as an anticipated outcome at the system level when conserved adaptive mechanisms in terminally differentiated, mechanically active tissues are perturbed. Therefore, a critical translational objective is to identify the juncture at which cardiomyocyte proteostasis signaling transitions from adaptive compensation to detrimental remodeling and damage in cardiomyocytes. This distinction is vital for therapy, as strategies that modulate the intensity, duration, or tissue exposure, rather than simply inhibiting or activating a pathway, are more likely to preserve antitumor efficacy while minimizing the cardiac risk. This approach supports (i) the optimization of doses and schedules for agents active in proteostasis, (ii) the use of rational combinations that alleviate the stress burden on any single pathway, and (iii) the integration of cardioprotective cotherapies and monitoring systems into oncology practices.

**FIGURE 5:**
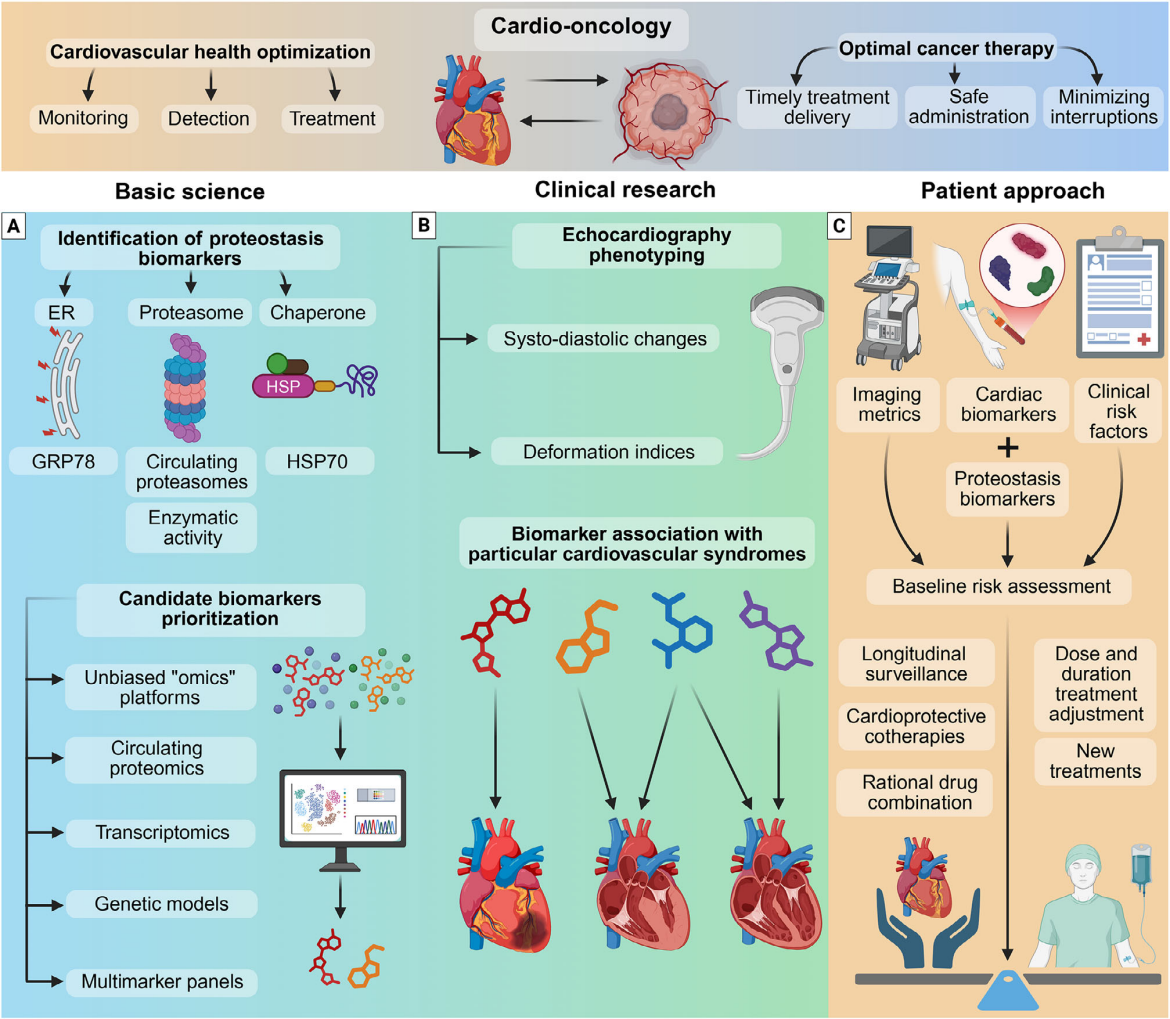
Comprehensive view of cardio‐oncology from research to clinical application. (A) Basic research is crucial for identifying new biomarkers and prioritizing them using various methods. (B) Combining echocardiographic parameters with biomarkers linked to specific cardiovascular conditions helps identify phenotypes that are prone to cardiotoxicity. (C) These findings facilitate a personalized, patient‐focused approach by accurately assessing the baseline cardiac risk using imaging metrics, biomarkers, and clinical data, aiding physicians in choosing customized anticancer treatments. Similarly, ongoing cardiac monitoring during anticancer therapy using these tools may optimize treatment plans through dose modifications, drug combinations, and cardioprotective co‐therapies, ultimately preserving cardiac function and treatment effectiveness. Created with BioRender.com.

Equally important is the recognition that disease‐ and therapy‐specific proteostasis signatures present opportunities for developing biomarkers and personalized risk assessments. Contemporary cardio‐oncology guidelines increasingly emphasize the integration of circulating biomarkers (such as troponins and natriuretic peptides), imaging metrics, and clinical risk factors to identify at‐risk patients and determine the intensity of monitoring (Figure 5C). Biomarker‐informed strategies are particularly advantageous for regimens targeting proteostasis, as myocardial damage may initially present as a subclinical stress phenotype characterized by ER stress/UPR engagement, autophagic imbalance, and gradual deterioration of protein quality control before any overt dysfunction becomes apparent.

In conclusion, while cancer and CVDs share fundamental proteostasis machinery, they differ significantly in their utilization, tolerance, and resolution. Tumor cells exploit proteostasis flexibility to survive and adapt to chronic stress, whereas cardiomyocytes depend on proteostasis fidelity to maintain their structure and function over extended periods. This asymmetry elucidates why therapeutic manipulation of proteostasis often results in contrasting outcomes across different tissues and provides a coherent framework for applying proteostasis biology in clinical decision‐making. In the subsequent section, we further explore this shared yet divergent model to examine therapeutic opportunities and challenges, focusing on drugs targeting proteostasis, the mechanistic causes of cardiotoxicity, and rational strategies for cardioprotective interventions.

## 5 Therapeutic Implications of Proteostasis Dysregulation at the Cancer–CVDs Interface

The primary translational challenge in proteostasis biology lies in the dual role of pathways that present therapeutic vulnerabilities in cancer while being essential for cardiovascular homeostasis. Consequently, interventions aimed at exploiting tumor reliance on protein quality control often function within a limited therapeutic window, where antitumor effectiveness and cardiovascular safety are closely associated. This issue is particularly pronounced in therapies targeting the proteasome, autophagy, ER stress responses, and molecular chaperone networks, the disruption of which can simultaneously induce tumor cell death and undermine myocardial resilience. This section explores the therapeutic opportunities and risks associated with shared proteostasis pathways, emphasizing the mechanistic basis of treatment‐related cardiotoxicity, strategies for cardioprotection and therapeutic repurposing, and the evolving framework of mechanism‐guided precision cardio‐oncology.

### 5.1 Proteostasis‐Targeting Therapies as Double‐Edged Swords

The understanding that malignant cells frequently persist and even thrive by enhancing their protein quality control systems has initiated a therapeutic trend aimed at inducing proteotoxic collapse as a strategy for cancer treatment. This approach extends beyond mere proteasome inhibition to encompass the entire proteostasis network of the cell. Pharmacological modulation of autophagy, disruption of UPR signaling, and interference with molecular chaperone functions, particularly HSP90, can destabilize tumor proteomes by depleting compensatory reserves and elevating stress levels beyond tolerable limits [93, 131, 153]. Notwithstanding, the very principle that renders these strategies effective against cancer also presents a significant risk to the cardiovascular system of the patients. The adult heart, predominantly composed of long‐lived, terminally differentiated cardiomyocytes, relies on continuous proteostasis to maintain sarcomeric structure, mitochondrial health, and excitation–contraction coupling over decades of mechanical activity. Consequently, systemic disruption of proteostasis not only induces “off‐target” toxicity but also challenges the fundamental homeostatic requirement of cardiac function, thereby constraining the therapeutic window for many proteostasis‐targeted treatments [20, 156].

PIs exemplify this paradox with a particular clinical precision. In plasma cell cancers, the therapeutic index of PIs is determined by a biologically logical vulnerability: the synthesis and secretion of immunoglobulins creates consistently high ER and proteotoxic stress, rendering myeloma cells unusually dependent on continuous proteasomal clearance to prevent the accumulation of lethal proteins [20, 157]. This dependency has led to the transformative efficacy of bortezomib in relapsed multiple myeloma, where proteasome inhibition has resulted in improved clinical outcomes compared with high‐dose dexamethasone and has established PIs as essential agents in contemporary myeloma treatment [157, 158]. However, the heart perceives proteasome inhibition from a fundamentally different perspective than other organs. In cardiomyocytes, inhibition of proteasomal degradation can impede the turnover of damaged contractile proteins, impair mitochondrial quality control, and exacerbate oxidative and inflammatory stress. These disturbances are often temporarily tolerated but become clinically significant when combined with age‐related declines or pre‐existing cardiovascular conditions [159, 160].

Reflecting this mechanistic susceptibility, cardiovascular adverse events (CVAEs) have become a hallmark of toxicity associated with irreversible proteasome inhibition, particularly with carfilzomib. Meta‐analyses of prospective trials have revealed significant rates of CVAEs with carfilzomib, such as hypertension and heart failure, with a notable proportion of patients experiencing severe events [159]. Concurrently, comprehensive analyses from Phase I–III trials confirm that Grade ≥3 hypertension, dyspnea, and cardiac failure are consistent indicators, even though fatal events do not significantly increase and treatment discontinuations are relatively rare. This highlights a pattern of manageable cardiovascular risk that is mechanistically linked, necessitating proactive monitoring and management of risk factors rather than attributing them to existing comorbidities [161]. Notably, the ENDEAVOR trial (comparing carfilzomib–dexamethasone with bortezomib–dexamethasone) demonstrated the superior antimyeloma effectiveness of carfilzomib while also clarifying the clinical trade‐offs: enhanced proteasome inhibition can achieve greater tumor control but requires increased cardiovascular monitoring [162, 163, 164].

In the broader context of cardio‐oncology, the concepts of “tumor dependence” and “cardiac fragility” are interconnected when therapies target the shared proteostasis machinery. In tumors, the aim is to push the already stressed proteome beyond a critical point, leading to apoptosis or irreversible dysfunction of the tumor cells. In cardiomyocytes, the same stress can deplete reserves and trigger maladaptive remodeling, especially with cumulative dosing, concurrent cardiometabolic stressors, and underlying structural heart disease. This duality suggests a therapeutic approach in which PI‐associated cardiotoxicity is seen as a predictable outcome of proteostasis overload, to be mitigated through careful patient selection, initial cardiovascular assessment, biomarker and echocardiogram‐guided monitoring, and strategic cotherapies, rather than as an unpredictable side effect unrelated to the drug's primary action.

### 5.2 Mechanistic Basis of Anticancer Therapy‐Induced Cardiotoxicity

Extensive experimental and clinical evidence suggests that the cardiotoxicity associated with many modern anticancer therapies arises from increased proteostasis stress that exceeds the heart's adaptive capacity rather than from a random collection of unique “off‐target” effects. Treatments affecting proteostasis, such as PIs, autophagy modulators, agents engaging the UPR/ER stress, and drugs targeting chaperones, frequently result in a common set of downstream pathological events in cardiac muscle cells. These events include the accumulation of damaged or aggregation‐prone proteins, disruption of mitochondrial quality control, increased oxidative stress, instability in calcium handling, and maladaptive inflammatory signaling pathways [165, 166]. In terminally differentiated and mechanically demanding tissues, these disruptions are particularly harmful as they diminish the functional reserve long before any noticeable loss of cardiac muscle cells occurs.

Proteasome inhibition is an example of this mechanism. While PIs exert their antitumor effects by pushing proteome instability and proteotoxic stress beyond the tolerance of highly burdened cancer cells, the same process in cardiac muscle cells can impede the removal of damaged sarcomeric and regulatory proteins, disrupt mitochondrial balance, and exacerbate oxidative and inflammatory stress pathways that promote adverse remodeling [165, 166]. In this context, cardiovascular toxicity is not incidental; it is an anticipated consequence of inhibiting a fundamental proteolytic pathway in a tissue whose function depends on continuous, precise protein turnover.

A similar logic applies to autophagy modulation, where the heart's reliance on precisely regulated basal flux renders it susceptible to therapeutic perturbations. Autophagy inhibition may reduce stress tolerance by restricting organelle and aggregate clearance during periods of increased demand. Conversely, excessive or prolonged induction of autophagy can lead to a shift from adaptive recycling to energetic depletion and maladaptive remodeling processes. Notably, these risks are most pronounced under combination regimens that concurrently stress multiple proteostasis pathways (e.g., UPS combined with autophagy or ER stress combined with autophagy), thereby compressing the compensatory capacity and lowering the threshold for dysfunction.

ER stress and the UPR represent the third clinically significant convergence point. Several anticancer strategies directly induce ER stress as part of a proteotoxic‐kill strategy or indirectly increase ER burden by disrupting proteostasis [167]. When ER stress is transient, UPR signaling can be adaptive; however, when sustained, it can activate terminal programs that intersect with the inflammation, mitochondrial dysfunction, and apoptosis pathways [165]. This transition from adaptive to terminal UPR is particularly critical in cardiomyocytes, where prolonged UPR activation is linked to maladaptive remodeling and progression toward heart failure phenotypes.

Targeted therapies that disrupt molecular chaperone networks further exemplify how cardiotoxicity can be mechanistically “on‐pathway.” Pharmacological inhibition of HSP90 destabilizes multiple oncogenic client proteins simultaneously, presenting an attractive poly node anticancer strategy. However, it also undermines a central buffering system that maintains the conformational stability of key signaling and structural proteins under stress [139, 153]. In the myocardium, where chaperone activity is crucial for maintaining calcium‐handling fidelity, mitochondrial integrity, and overall proteome stability, chaperone inhibition can exacerbate ER stress and lower the threshold for dysfunction during concurrent hemodynamic or metabolic strains [168, 169].

Altogether, these observations support a unifying interpretation: across various drug classes, anticancer therapy‐induced cardiotoxicity frequently emerges as a mechanistically predictable outcome of perturbing the shared proteostasis circuitry in tissues with limited tolerance for sustained proteome instability. Recognizing cardiotoxicity as an emergent consequence of proteostasis imbalance shifts translational priorities from reactive management of “side effects” to proactive control of stress dose (intensity × duration), rational combination design, and cardioprotective strategies that preserve myocardial proteostasis reserve while maintaining antitumor efficacy.

### 5.3 Opportunities For Therapeutic Repurposing and Cardioprotective Costrategies

**FIGURE 6:**
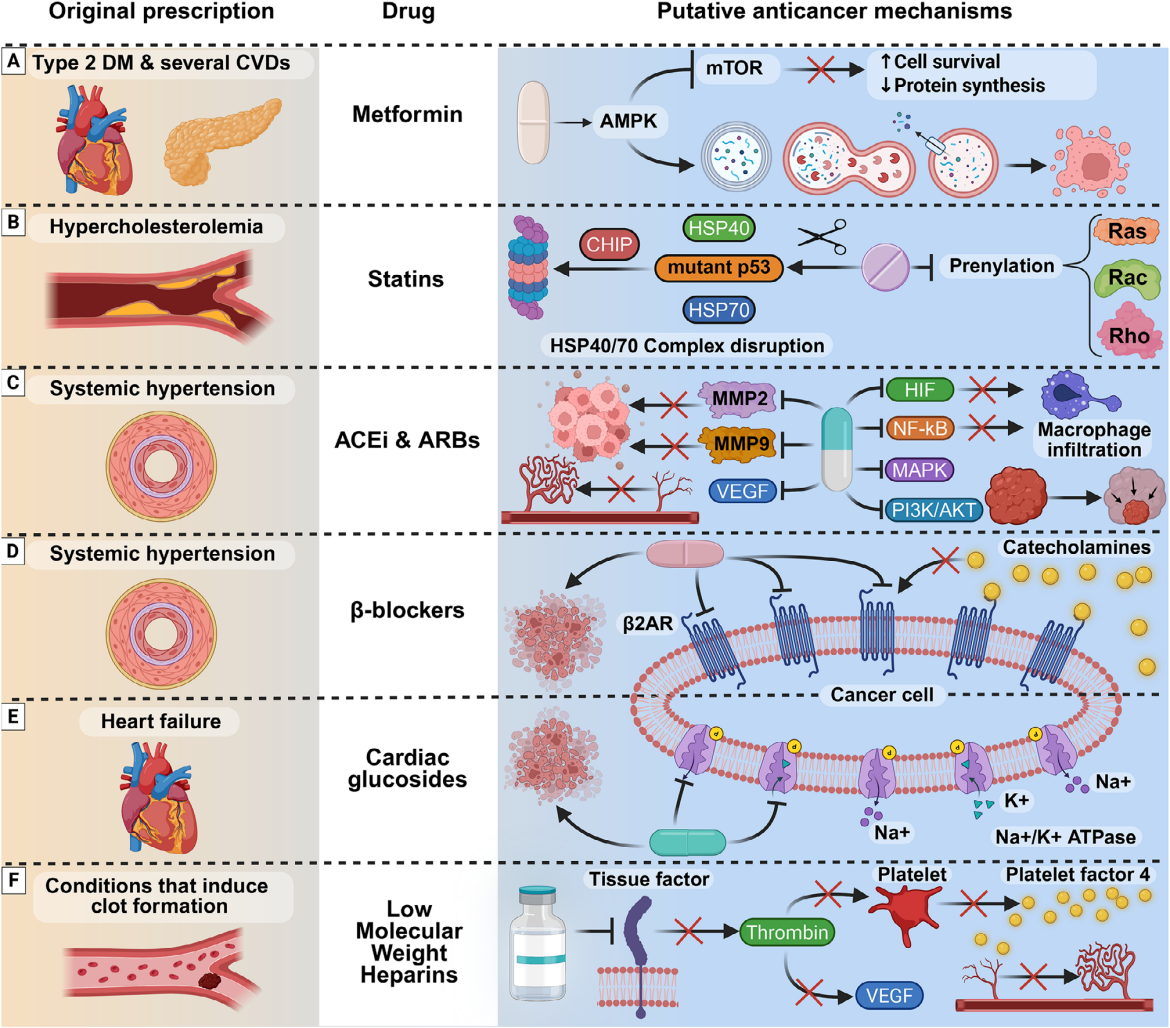
Transition from cardiology to oncology: repurposing cardiovascular drugs for cancer treatment. The biological similarities between cancer and cardiovascular diseases (CVDs) justify the repurposing of cardiovascular drugs for the treatment of cancer. (A) Metformin influences the AMPK–mTOR pathway, affecting autophagy and protein synthesis via AMPK‐related actions. (B) Statins interfere with the protein complexes that protect mutant p53 and prevent the prenylation of several oncogenic proteins. (C) Angiotensin‐converting enzyme inhibitors and Angiotensin II receptor blockers work by decreasing inflammatory cell infiltration, angiogenesis, and metalloproteinase activity. (D) and (E) Beta‐blockers and cardiac glycosides target β2‐adrenergic receptors and Na^+^/K^+^‐ATPase on tumor cell membranes, respectively, thereby inhibiting tumor growth. (F) Low‐molecular‐weight heparins have been shown to inhibit angiogenesis via tissue factor‐mediated downstream signaling. *Abbreviations*: type 2 diabetes mellitus (Type 2 DM); metalloproteinase (MMP); vascular endothelial growth factor (VEGF); nuclear factor‐κB (NF‐κB); mitogen‐activated protein kinase (MAPK); hypoxia‐inducible factor (HIF); angiotensin‐converting enzyme inhibitors (ACEi); Angiotensin II receptor blockers (ARBs); β2‐adrenergic receptors (β2AR); protein kinase B (AKT); and phosphatidylinositol 3‐kinase (PI3K). Created with BioRender.com.

A proteostasis‐centered framework not only elucidates cardiotoxic risks but also identifies practical opportunities for therapeutic advancements through drug repurposing and cardioprotective strategies. Many commonly used cardiovascular drug classes interact directly or indirectly with proteostasis control points, including autophagy regulation, stress signaling, inflammatory remodeling, and protein quality control. This interaction implies that certain drugs may influence tumor biology in specific contexts while simultaneously enhancing the cardiovascular resilience. The repurposing of cardiovascular drugs for anticancer purposes is actively being investigated, although the results vary across different tumor types and study designs [170, 171]. β‐Adrenergic blockade is particularly noteworthy because of the established role of adrenergic signaling in stress adaptation and immune modulation. However, clinical findings are inconsistent, with recent meta‐analyses indicating potential survival or progression benefits in certain situations but no universal effect across all cancer types [172, 173, 174]. Statins have also attracted significant interest; beyond their lipid‐lowering effects, they can affect membrane‐associated signaling and inflammatory responses. Several meta‐analyses, particularly those focusing on advanced‐stage disease or breast cancer cohorts, have found associations with improved cancer outcomes, although effect sizes differ, and residual confounding remains a concern [175, 176, 177]. Regarding renin–angiotensin system blockade, observational meta‐analyses have attempted to resolve ongoing debates about cancer risk and outcomes of cancer. The most comprehensive syntheses highlight the inconsistencies across studies and emphasize the need for careful causal inference rather than broad generalizations [178, 179, 180]. Overall, these findings argue against a “class‐wide anticancer effect” but support the notion that modulating host stress responses, including proteostasis‐linked programs, could be therapeutically relevant in specific biological and clinical contexts, as summarized in Figure 6 [181, 182, 183, 184, 185, 186, 187, 188, 189].

On the other hand, cardioprotective cotherapies offer a more feasible approach to separate the antitumor effects from cardiac damage [8, 190]. Theoretically, interventions that either maintain or restore myocardial proteostasis by ensuring autophagic flux remains within a beneficial range, enhancing chaperone function, or reducing maladaptive ER stress and excessive UPR activation could boost cardiac stress resilience without necessarily protecting tumor cells, especially when the timing and exposure are carefully managed. Preclinical studies across proteostasis areas support this rationale: minor variations in stress intensity or duration can dictate whether adaptive responses remain beneficial or become harmful, suggesting that the therapeutic goal often involves modulating stress doses rather than inhibiting pathways. In clinical settings, a similar concept has already been integrated into cardio‐oncology through strategies that adjust schedules and exposures [191]. Meta‐analyses indicate that traditional cardiovascular drugs, particularly β‐blockers and ACEi/ARBs, are effective in preventing or reducing the decline in left ventricular function during anthracycline‐ or trastuzumab‐based treatments, a strategy that can be seen as preserving the reserve against proteotoxic and inflammatory stress [192, 193]. Furthermore, targeted delivery to specific tissues and exposures is an undervalued method for preserving proteostasis. Liposomal anthracycline formulations decrease myocardial exposure and consistently show reduced cardiotoxicity while maintaining antitumor efficacy in various contexts [194, 195]. These strategies, along with the temporal separation of stress‐inducing treatments from cardioprotective measures and the creation of tissue‐specific delivery systems, offer a viable route for enhancing the therapeutic indices of proteostasis‐focused regimens.

Overall, this evidence suggests that the most effective translational approach is not to avoid targeting proteostasis but to develop selectivity in terms of timing, tissue, and stress dosage using repurposed host‐directed therapies and exposure‐modifying strategies to sustain myocardial proteostasis while maintaining tumor‐targeted proteotoxic stress.

### 5.4 Toward Mechanism‐Guided Precision Cardio‐Oncology

Incorporating proteostasis biology into therapeutic decision‐making could transform cardio‐oncology from managing toxicity reactively to anticipating risks based on their mechanisms. Instead of viewing “cardiotoxicity” as a single phenotype, a proteostasis‐focused approach aligns each anticancer treatment with the specific proteostasis nodes it affects, such as proteasomal flux, autophagic capacity, ER stress/UPR signaling, and chaperone buffering, along with the downstream failure modes that are likely to occur in cardiomyocytes (including protein aggregation, mitochondrial dysfunction, oxidative stress amplification, calcium‐handling instability, and inflammatory remodeling). This mechanistic mapping is actionable in clinical settings as it elucidates why certain drug classes are consistently associated with specific cardiovascular syndromes and offers a logical foundation for choosing baseline phenotyping, monitoring intensity, and cotherapeutic support (Figure 5B).

The second, and arguably crucial, component of precision cardio‐oncology is a biomarker strategy that aligns with the underlying mechanism of disease rather than just late‐stage dysfunction. Current guidelines highlight the importance of troponins, natriuretic peptides, and imaging‐derived deformation measures (especially global longitudinal strain [GLS]) for early detection of cardiac dysfunction related to cancer therapy and for increasing monitoring when subclinical injury is suspected [7, 8, 196, 197]. Although these tools are not specific to proteostasis, they embody a key concept inherent in proteostasis biology: clinically significant injury often starts as a stress phenotype (characterized by reduced reserve, altered energetics, and subtle contractile impairment) before overt systolic dysfunction becomes apparent.

Biomarkers focused on proteostasis are emerging as promising candidates for enhancing mechanistic resolution and potentially facilitating earlier and more specific detection [198]. Circulating or extracellular chaperones, including proteins from the HSP70 family, have been investigated as indicators of stress in both cancer and CVDs. These chaperones may eventually assist in monitoring therapy response or risk enrichment in specific settings, although challenges such as standardization, tissue attribution, and systemic inflammation confounding remain significant limitations [199, 200, 201]. Concurrently, ER stress‐associated proteins, such as GRP78/BiP (HSPA5), and related stress–response signatures have been extensively researched as markers of tumor stress adaptation and therapeutic resistance. These proteins are being evaluated more broadly as components of stress‐phenotyping approaches [202, 203, 204]. Additionally, unbiased “omics” platforms, including circulating proteomics, transcriptomics, and multimarker panels, are increasingly employed to prioritize candidate biomarkers linked to myocardial stress biology and to refine risk prediction beyond single‐analyte paradigms, offering a promising discovery framework for proteostasis‐linked signatures [205, 206, 207, 208, 209] (Figure 5A).

Ultimately, mechanism‐guided precision cardio‐oncology requires the alignment of antitumor efficacy with cardiac resilience by understanding tissue‐specific stress thresholds and tolerance windows. The clinical implications are both straightforward and significant: therapies targeting proteostasis occupy a central and inherently precarious position in contemporary oncology. Their effectiveness arises from exploiting tumor reliance on adaptive protein quality control, whereas their toxicity reflects the myocardium's vulnerability to sustained proteostasis stress. Recognizing this shared mechanistic basis redefines cardiotoxicity from an unavoidable complication to a biologically interpretable and potentially preventable outcome. This outcome depends on managing the cumulative stress dose, tailoring surveillance to the mechanism, and implementing cardioprotective costrategies when the reserve is limited.

## 6 Clinical and Translational Perspectives

Perhaps the most significant implication of proteostasis biology is its challenge to the traditional assumption in modern oncology: the separation of therapeutic efficacy and toxicity. Emerging evidence indicates that these phenomena may manifest differently in the same underlying biological process. Proteotoxic stress responsible for tumor collapse is often mechanistically linked to stress that compromises myocardial resilience, thereby connecting cancer control and cardiovascular injury through a shared proteostasis framework. From this perspective, cardiotoxicity is not just a side effect to be identified postoccurrence but a biologically foreseeable outcome of how tissues individually perceive, manage, and ultimately fail to handle therapeutic stress. This perspective shift has extensive implications for translational research and clinical practice. This section explores how insights from preclinical models, clinical trials, and real‐world cardio‐oncology converge to redefine risk assessment, biomarker development, and therapeutic decision‐making. This convergence lays the groundwork for a precision medicine framework aimed at not only effectively treating cancer but also preserving cardiovascular resilience.

### 6.1 Preclinical Evidence Linking Proteostasis Dysregulation to Cardio‐Oncology Outcomes

Preclinical models have been instrumental in elucidating the connection between proteostasis disruption and the interplay between tumor management and cardiac vulnerability, primarily through predictable biological mechanisms. In murine cancer therapy models, pharmacological agents that destabilize the tumor proteome, particularly PIs, as well as treatments that enhance ER stress/UPR signaling or disrupt compensatory clearance pathways consistently exhibit antitumor effects while inducing cardiac conditions associated with protein quality control failure. These conditions include reduced contractility, mitochondrial damage, increased oxidative stress, and maladaptive remodeling with fibrosis [210, 211]. These findings strongly indicate that cardiotoxicity in many proteostasis‐targeted treatments is not merely a nonspecific consequence of systemic illness but a direct result of imposing a proteotoxic burden on tissues with limited reserve capacity.

Proteasome inhibition provides the most definitive experimental evidence for this principle. In a well‐characterized mouse model, the administration of carfilzomib resulted in left ventricular dysfunction accompanied by molecular markers indicative of impaired cardiac proteostasis and stress signaling. However, cotreatment with metformin improved functional outcomes and mitigated injury‐related pathways, exemplifying how proteostasis‐focused mechanisms can be experimentally modulated to alter cardiac outcomes without compromising the antitumor efficacy of treatment [210]. Furthermore, aging, a significant clinical risk factor for therapy‐related cardiotoxicity, has been shown to exacerbate carfilzomib‐induced cardiac dysfunction in vivo, underscoring that cardiac damage occurs when therapy‐induced stress exceeds the age‐ and disease‐conditioned tolerance threshold of myocardial proteostasis [211].

Genetic models provide valuable insights by demonstrating how myocardial vulnerability can be modified through the targeted manipulation of specific proteostasis pathways. For example, when autophagy machinery is specifically disrupted in cardiomyocytes, it becomes apparent that the loss of proteostasis reserve compromises functional capacity under stress conditions. Although baseline phenotypes may appear mild or delayed, stress exposure reveals compromised reserve, mitochondrial dysfunction, and calcium handling defects, potentially increasing the heart's susceptibility to additional proteotoxic challenges from anticancer treatments [81, 212]. Similarly, interference with UPR signaling components redefines the balance between adaptive compensation and decompensation. A deficiency in PERK significantly exacerbates remodeling and heart failure induced by pressure overload, indicating that endogenous UPR signaling can protect the heart within a specific stress range. Consequently, therapies that increase or misregulate ER stress may drive cardiomyocytes toward maladaptive and failure‐prone pathways [212]. The study of ATF6 biology offers a particularly insightful example: transgenic activation of ATF6‐dependent pathways in cardiomyocytes reduces ischemia/reperfusion injury, while loss‐of‐function models demonstrate that endogenous ATF6 helps limit stress‐related damage. Collectively, these findings illustrate how targeted enhancement of adaptive proteostasis mechanisms can alter outcomes without the need to inhibit entire pathways [213, 214]. Genetic studies focusing on the UPS further reinforce this mechanistic connection, as E3 ubiquitin ligases and ubiquitin‐related signaling can influence inflammatory remodeling and cell death during cardiac stress. A recent study identified an E3 ligase axis (MARCH2) that limits inflammasome activation and pyroptosis during ischemia/reperfusion, providing a clear example of how ubiquitin‐mediated proteostasis control intersects with inflammatory cell death processes relevant to cardiotoxic phenotypes and postinjury remodeling [215]. Collectively, these genetic and pharmacological findings suggest a common conclusion: cardiac outcomes are less determined by which proteostasis pathway is disrupted and more by whether the extent and duration of disruption exceed the compensatory reserve.

Conversely, tumor models underscore the bidirectional nature of this systemic issue. Tumors that enhance proteostasis capacity through HSF1‐driven chaperone programs and related buffering networks exhibit increased stress tolerance and resistance to therapy in vivo, indicating that proteostasis reserve is a selectable trait influencing oncologic response [216]. Similarly, the upregulation of ER chaperones such as GRP78 is consistently associated with chemoresistant phenotypes and decreased sensitivity to therapy‐induced stress, further emphasizing that the same adaptive mechanisms that enhance tumor fitness can create collateral vulnerability in the heart when activated systemically [203].

Overall, these preclinical findings establish proteostasis as a central mechanism linking anticancer effectiveness to cardiovascular risk: tumor control is often achieved by pushing proteotoxic stress beyond a lethal threshold, while cardiotoxicity arises when the same stress surpasses the myocardium's limited tolerance. This paradigm provides a logical foundation for mechanism‐informed modeling, cotherapy design, and biomarker development in translational cardio‐oncology research.

### 6.2 Clinical Trials Targeting Proteostasis Pathways: Efficacy and Cardiac Risk

Over the past decade, the clinical implementation of strategies targeting proteostasis has progressed significantly, particularly in hematologic oncology, where cancer cells are heavily dependent on protein quality control mechanisms. PIs represent the most advanced clinical application of this approach [165]. Bortezomib exemplifies this concept by exploiting the increased proteotoxic stress in plasma cell cancers, resulting in sustained clinical benefits in relapsed multiple myeloma and becoming a crucial component of contemporary combination treatments [157]. Similarly, second‐generation irreversible inhibitors, such as carfilzomib, have demonstrated enhanced antimyeloma efficacy in randomized Phase III trials, confirming that more profound proteasome inhibition can lead to improved cancer outcomes [162]. Oral ixazomib has further expanded the PI field, enabling continuous proteostasis targeting in outpatient settings and proving effective in combination therapy [217, 218, 219].

However, clinical experience has underscored the cardiovascular risks associated with the systemic disruption of proteostasis [165]. Across various trials and postmarketing observations, PIs, particularly carfilzomib, have been consistently associated with higher rates of CVAEs, including hypertension, heart failure, arrhythmias, and ischemic conditions [159, 220]. Meta‐analyses indicate that the incidence of clinically significant CVAEs is not uncommon, and mechanistic interpretations increasingly view these toxicities as direct consequences of proteostasis overload in the heart, which has limited stress tolerance [159]. These findings highlight a critical translational challenge: treatments that induce proteotoxic crises in tumors may simultaneously compromise cardiac reserve, especially in older patients or those with preexisting cardiovascular conditions.

Beyond the proteasome, therapeutic modulation of autophagy and ER stress‐induced UPR signaling is being actively explored in clinical settings. Chloroquine and hydroxychloroquine are repurposed lysosomotropic agents that disrupt autophagosome‐lysosome fusion and have been extensively tested as chemosensitizers in early phase clinical oncology trials [221, 222]. While inhibiting autophagy is conceptually appealing for destabilizing tumor stress tolerance, these agents are known to pose cardiovascular risks at higher doses, including QT prolongation and cardiomyopathy, underscoring the need for careful dosing and monitoring when used in combination with other agents [223].

Targeting the UPR and chaperone networks presents additional therapeutic opportunities. Small‐molecule inhibitors of IRE1 RNase activity, PERK signaling, and HSP90 function are currently undergoing Phase I/II evaluations for solid and hematologic cancers [121, 224, 225]. Although these approaches are mechanistically promising, aiming to disrupt adaptive proteostasis buffering in tumors, systematic cardiovascular monitoring is often inconsistent in early phase trials, and cardiac endpoints are frequently underpowered or inadequately assessed in these studies. This gap underscores the urgent need for trial designs aligned with mechanisms that incorporate baseline cardiovascular phenotyping, biomarker monitoring, and prospective definitions of proteostasis‐related cardiotoxicity.

Accordingly, Table 1 provides a summary of representative ongoing and completed clinical trials of proteostasis‐targeting therapies, categorized by pathway, molecular target, cancer type, trial phase, cardiovascular endpoints, and their current status. This synthesis offers a practical framework for aligning therapeutic innovations with cardiovascular safety, as proteostasis‐directed strategies continue to evolve in oncology.

**TABLE 1:** Overview of selected clinical and preclinical studies on drugs affecting proteostasis in cancer and cardiovascular diseases. This table summarizes studies and trials assessing the therapeutic potential of drugs that either directly influence the core proteostasis machinery or indirectly affect pathways related to proteostasis in cancer and cardiovascular diseases. Interventions are classified according to their primary mechanistic focus and the stage of translational development. The table highlights not only clinical trials but also emerging preclinical strategies that could influence future therapeutic approaches.

Intervention (reference)	Proteostasis axis	Disease context	Trial identifier	Development stage	Description and translational relevance
A. Direct targeting of proteostasis machinery in cancer					
ARV‐110 (bavdegalutamide) [226]	Ubiquitin–proteasome system (targeted protein degradation)	Metastatic castration‐resistant prostate cancer	NCT03888612	Phase I/II (completed)	A first‐in‐class PROTAC degrader designed to selectively eliminate androgen receptor protein. Clinical evaluation has demonstrated activity in heavily pretreated patients, establishing proof of concept for targeted protein degradation in resistant prostate cancers.
ARV‐471 (vepdegestrand) [227, 228]	Ubiquitin–proteasome system (targeted protein degradation)	ER‐positive/HER2‐negative advanced metastatic breast cancer	NCT05654623	Phase III (ongoing)	An estrogen receptor‐directed PROTAC that induces proteasomal degradation rather than receptor blockade. The study compares ARV‐471 vs. fulvestrant, with ARV‐471 showing an improvement in progression‐free survival, highlighting a paradigm shift from inhibition to degradation‐based targeting.
Bortezomib [229]	Proteasome inhibition	Multiple myeloma	NCT00522392	Phase III (terminated due to slow accrual)	A reversible inhibitor of the 26S proteasome that induces proteotoxic stress and apoptosis in malignant plasma cells. Although this specific trial was terminated, bortezomib remains a cornerstone therapy and prototypical example of proteostasis‐targeted intervention.
Carfilzomib [230]	Proteasome inhibition	Relapsed or refractory multiple myeloma	NCT01302392	Phase III (completed)	An irreversible proteasome inhibitor with enhanced specificity compared with earlier agents. Its clinical efficacy underscores the dependence of myeloma cells on proteasomal function, whereas its cardiotoxic profile exemplifies the vulnerability of cardiac proteostasis.
Ixazomib [231]	Proteasome inhibition	Relapsed or refractory multiple myeloma	NCT02206425	Phase I/II (completed)	An orally bioavailable proteasome inhibitor that expands the therapeutic landscape of proteasome‐targeting strategies. Its pharmacological profile allows for more flexible dosing regimens while maintaining proteotoxic pressure in the tumor cells.
Ganetespib (STA‐9090) [232]	Chaperone inhibitor (HSP90 inhibition)	Advanced non‐small cell lung cancer	NCT01348126	Phase II (terminated)	A small‐molecule inhibitor of HSP90 that simultaneously destabilizes multiple oncogenic client proteins. This study explored monotherapy and combination regimens. By targeting chaperone‐dependent protein folding, ganetespib disrupts tumor signaling networks at the system level.
Ganetespib (STA‐9090) [233]	Chaperone inhibitor (HSP90 inhibition)	Metastatic hormone‐resistant prostate cancer	NCT01270880	Phase II (completed)	Evaluated in a distinct disease context, this study highlights the broad applicability of chaperone inhibition across tumor types driven by proteostasis‐dependent oncogenic networks, although it showed minimal clinical activity in this trial.
VER‐155008 [234, 235]	Chaperone inhibitor (HSP70 inhibition)	Cancer (experimental models)	—	Preclinical	A prototype HSP70 inhibitor that disrupts the protein‐folding networks required for tumor survival. HSP70 inhibition sensitizes cancer cells to bortezomib. Although not yet in clinical trials, it represents a promising approach for targeting the proteostasis buffering capacity in cancer.
LYTAC‐based approaches [236, 237, 238]	Lysosomal targeting pathways	Cancer (experimental models)	—	Preclinical	Lysosome‐targeting chimeras (LYTACs) extend targeted degradation strategies to extracellular and membrane proteins. These approaches expand the scope of proteostasis manipulation beyond the intracellular proteasomal pathways.
B. Indirect modulation of proteostasis in cardiovascular disease and cardio‐oncology					
Dapagliflozin [239]	Autophagy and cellular stress adaptation	Heart failure with reduced ejection fraction	NCT03036124	Phase III (completed)	A sodium‐glucose cotransporter 2 inhibitor that improves cardiovascular outcomes, in part, through the modulation of cellular stress responses and enhancement of autophagic flux. This represents an indirect but clinically validated link between metabolic therapy and proteostasis regulation.
Spermidine^a^	Autophagy induction	Arterial hypertension and cardiovascular aging	NCT04405388	Interventional study (closed: unknown status)	A naturally occurring polyamine that promotes autophagy and cellular resilience. Clinical investigations have focused on its effects on vascular function and systemic aging, with implications for the maintenance of proteostasis.
Spermidine^a^	Autophagy induction	Heart failure with preserved ejection fraction	NCT05128331	Interventional study (closed: unknown status)	Evaluates whether spermidine supplementation improves cardiovascular and metabolic responses to exercise training. Highlights the emerging role of autophagy modulation in the cardiac functional reserve.
Spermidine [240]	Autophagy induction	Elderly patients with coronary artery disease	NCT06186102	Interventional study (ongoing)	A long‐term supplementation study was designed to assess the impact of enhanced autophagic activity on cardiovascular health in aging populations. This study provide insights into the decline in proteostasis as a determinant of cardiovascular vulnerability.
BGP‐15 [241, 242, 243, 244]	Chaperone and stress–response modulation	Cardiovascular disease (preclinical emphasis)	—	Preclinical/exploratory	A small molecule that enhances cellular stress tolerance and exhibits chaperone activity. Although clinical cardiovascular data remain limited, preclinical studies suggest the potential for improving proteostasis resilience under cardiac stress conditions.

^a^ *Data sources*—clinical registration website.

### 6.3 Cardio‐Oncology Case Examples and Emerging Clinical Patterns

Specialized cardio‐oncology programs have increasingly identified distinct clinical patterns associated with proteostasis‐related vulnerabilities, particularly in patients undergoing treatment with PIs [165]. A prevalent observation is the early functional alteration that precedes overt systolic failure, which is often characterized by diastolic irregularities and subtle myocardial damage. These can be detected through advanced echocardiographic deformation imaging techniques, such as GLS, and circulating biomarkers [7, 245, 246]. Notably, detailed echocardiographic evaluations during carfilzomib treatment have demonstrated that systo‐diastolic changes and deformation metrics can manifest early in the treatment process. This reinforces the clinical perception that myocardial stress linked to PIs can be identified while the ejection fraction remains intact [245, 246]. Further evidence suggests that initial diastolic dysfunction parameters, such as *E*/*e*′ and septal *e*′, may predict future severe CVAEs, supporting the notion that limited pre‐existing reserve affects the heart's response to proteostasis stress [245] (Figure 5B).

The second significant pattern is the potential for partial reversibility when myocardial stress is detected early, and treatment is promptly adjusted. Observations from real‐world cases and institutional experiences indicate that modifying the dose, altering the schedule, or temporarily discontinuing carfilzomib often results in symptom improvement or resolution in a substantial number of patients with similar symptoms. In some cases, this allows for cautious reintroduction at a lower dose and/or with modified infusion parameters [247, 248]. This clinical response aligns with the underlying mechanisms, as proteostasis stress can cause functional impairment through reversible cellular disruptions, such as impaired protein turnover, mitochondrial stress, and redox imbalance, before irreversible remodeling or loss of cardiomyocytes becomes the predominant process. The practical implication is that cardiotoxicity from PIs is frequently not a sudden binary occurrence but a trajectory that can be redirected if identified at the “subclinical stress phenotype” stage.

Finally, cardio‐oncology clinics consistently observe considerable variability in cardiac tolerance to proteostasis‐active treatments, which cannot be fully explained by age, baseline ejection fraction, or traditional cardiovascular risk factors alone. Emerging insights from the cardio‐oncology genetics literature suggest a role for genetic and epigenetic factors that affect susceptibility to therapy‐related myocardial injury, including variants that influence oxidative stress management, inflammatory signaling, and stress response/protein quality control pathways [249, 250, 251]. Although the field remains diverse in terms of study design and replication strength, this variability strongly supports the need for mechanism‐based risk stratification. This approach should integrate baseline functional reserve, including diastolic indices and GLS, biomarker monitoring, and, in the future, validated genetic/omic predictors, rather than assuming a uniform risk of cardiotoxicity across all patient populations.

### 6.4 Biomarkers of Proteostasis Imbalance and Precision Medicine Implications

The identification of biomarkers that indicate disruptions in proteostasis, rather than merely signaling late‐stage mechanical failure, offers substantial translational opportunities in cardio‐oncology. Theoretically, therapies targeting proteostasis and various proteotoxic anticancer treatments induce a myocardial “stress phenotype” that can be detected prior to irreversible remodeling or a significant decline in left ventricular ejection fraction. Current consensus guidelines recommend combining circulating biomarkers, particularly cardiac troponins and natriuretic peptides, with imaging techniques, such as myocardial strain, for initial risk assessment and ongoing monitoring. This strategy is advocated because subclinical damage may occur before symptoms manifest, and early intervention is most effective when stress remains reversible [7, 8, 196, 197]. Within this broader framework, candidates focusing on proteostasis are emerging at various mechanistic levels.

#### 6.4.1 ER Stress/UPR‐Related Markers

ER chaperones, notably GRP78, are integral to the UPR and have been extensively studied in the context of cancer stress adaptation and therapy resistance in cancer cells [202, 203]. In cardiovascular research, experimental evidence suggests a causal relationship between GRP78‐mediated buffering and susceptibility to chemotherapy‐induced damage in the heart. For example, doxorubicin can disrupt protective ER stress responses, and restoring GRP78 expression reduces ER stress‐related cell death in preclinical models [218]. These findings support the translational hypothesis that circulating or stress‐inducible ER stress markers could enhance early risk assessment or detect evolving injury during proteostasis‐active treatments, although clinical standardization and tissue attribution remain significant challenges (Figure 5A).

#### 6.4.2 Proteasome‐Related Signatures

Circulating proteasomes and their enzymatic activity have been proposed as measurable systemic indicators of proteostasis, with historical observations of elevated circulating proteasome levels in malignancies and ongoing discussions on reliably quantifying their activity and concentration for biomarker applications [252, 253]. Concurrently, clinical cardio‐oncology experience with PIs, supported by mechanistic analyses, strongly suggests that myocardial vulnerability is linked to the proteostasis reserve. This implies that proteasome‐related signatures may eventually help define a “susceptibility phenotype” when combined with traditional cardiac biomarkers and imaging [165] (Figure 5A).

#### 6.4.3 Chaperone‐Associated Signals

HSPs, particularly members of the HSP70 family, are recognized stress reporters with established extracellular roles in cancer, including their potential use as circulating biomarkers and as immunomodulatory signals [199]. Although their specificity for myocardial injury is limited and systemic inflammation presents a significant confounding factor, the broader principle remains: stress‐inducible proteostasis reporters may be most effective when used as part of multimarker panels rather than as stand‐alone predictors (Figure 5A).

Importantly, the immediate clinical value of proteostasis biomarkers is unlikely to stem from replacing troponin/BNP or imaging but rather from enhancing mechanistic understanding. They can aid clinicians in distinguishing which therapies predominantly cause ER stress versus impaired protein clearance and which patients are nearing their tolerance limits. Reviews focusing on cancer therapy‐related cardiac dysfunction increasingly emphasize this direction, highlighting that next‐generation biomarkers should be detectable before clinical toxicity occurs and, ideally, should reflect the underlying mechanism rather than a generic injury signal [196, 206, 254].

The implications of precision medicine are evident: integrating biomarkers associated with proteostasis with echocardiography/strain and clinical risk factors may enable the customization of monitoring intensity, the earlier initiation of cardioprotective interventions, and the potential adjustment of dosing or scheduling of proteostasis‐targeted therapies based on biomarkers. This strategy seeks to preserve myocardial reserve while ensuring effective antitumor activity [7, 8, 196, 206]. More broadly, both clinical and translational evidence increasingly suggest that the dysregulation of proteostasis is a critical factor affecting therapeutic success and cardiovascular side effects. Therefore, incorporating proteostasis biology into the design and monitoring of trials offers an opportunity for proactive rather than reactive management of cardiotoxicity, facilitating precision strategies that protect cardiac health while maintaining the efficacy of cancer treatments [8, 165, 206] (Figure 5C).

## 7 Conclusion and Future Perspectives

CVDs and cancer, which were historically viewed as distinct clinical entities, are now acknowledged to share substantial molecular similarities, particularly in the regulation of proteostasis. This review underscores that the UPS, autophagy–lysosome pathway, UPR, and molecular chaperone networks collectively constitute a conserved adaptive system that facilitates cellular survival under proteotoxic stress. While these pathways are essential for maintaining cardiac proteome integrity amidst prolonged mechanical stress, malignant cells frequently exploit them to promote unchecked growth, metabolic adaptability, and treatment resistance. This imbalance in reliance and tolerance provides a unified mechanistic explanation for the frequent clinical overlap between the success of cancer treatment and cardiovascular side effects.

A critical insight from this analysis is that proteostasis is neither inherently protective nor harmful to the organism. Instead, it operates within context‐specific limits determined by cellular identity, stress level, and timing. Tumor cells augment their proteostasis capacity by enhancing chaperone buffering, proteasomal activity, autophagic recycling, and sustained engagement of the UPR to withstand chronic stress and therapeutic challenges. Conversely, cardiomyocytes function within a constrained adaptive range that is optimized for long‐term structural stability rather than immediate adaptability. When treatments exceed this range, cardiac dysfunction emerges as a predictable biological outcome, rather than an unforeseen side effect. Understanding this concept redefines cardiotoxicity as a mechanistically comprehensible result of shared stress–response mechanisms rather than as a secondary complication.

Proteostasis biology offers a valuable framework for advancing precision cardio‐oncology research. Therapeutic designs informed by mechanisms and tissue‐specific proteostasis capacities may enable the selective destabilization of the tumor proteome while preserving myocardial resilience. Integrating proteostasis‐related biomarkers with functional imaging, traditional cardiac injury markers, and novel genetic modifiers holds potential for early risk assessment, dynamic monitoring, and personalized therapy. Such approaches require close collaboration between oncologists, cardiologists, and basic scientists to translate system‐level insights into practical and clinical protocols.

Looking forward, research should advance beyond single‐pathway interventions to network‐informed modulation of proteostasis, employing temporal control, tissue specificity, and rational combination strategies to optimize the therapeutic outcomes. As cancer survival rates continue to improve, ensuring long‐term cardiovascular health will become an increasingly vital component of comprehensive cancer care in the future. By positioning proteostasis at the intersection of mechanism, therapy, and clinical outcomes, this review highlights its potential to guide the next generation of integrative, patient‐focused approaches in cardio‐oncology and in cardiovascular medicine.

## Author Contributions

N.F. and A.Y.R. conceived and presented the ideas. J.E.G.T. and A.Y.R. performed the literature search wrote the manuscript. J.E.G.T. created the figures in this manuscript. N.F. and A.Y.R. provided the financial support. All authors have reviewed and approved this article.

## Ethics Statement

The authors have nothing to report.

## Conflicts of Interest

The authors declare no conflicts of interest.

## Data Availability

The authors have nothing to report.

## References

[1] N. S. Wilcox , U. Amit , J. B. Reibel , E. Berlin , K. Howell , and B. Ky , “Cardiovascular Disease and Cancer: Shared Risk Factors and Mechanisms,” Nature Reviews Cardiology 21 (2024): 617–631. 38600368 10.1038/s41569-024-01017-x PMC11324377

[2] R. J. Koene , A. E. Prizment , A. Blaes , and S. H. Konety , “Shared Risk Factors in Cardiovascular Disease and Cancer,” Circulation 133 (2016): 1104–1114. 26976915 10.1161/CIRCULATIONAHA.115.020406 PMC4800750

[3] C. Shu , H. Han , H. Li , et al., “Cancer Risk Subsequent to Cardiovascular Disease: A Prospective Population‐Based Study and Meta‐Analysis,” BMC Medicine [Electronic Resource] 23 (2025): 192. 40165228 10.1186/s12916-025-04013-1 PMC11959738

[4] P. Libby , “Mechanisms of Acute Coronary Syndromes and Their Implications for Therapy,” New England Journal of Medicine 368 (2013): 2004–2013. 23697515 10.1056/NEJMra1216063

[5] D. Hanahan and R. A. Weinberg , “Hallmarks of Cancer: The next Generation,” Cell 144 (2011): 646–674. 21376230 10.1016/j.cell.2011.02.013

[6] T. Hasin , Y. Gerber , S. A. Weston , et al., “Heart Failure After Myocardial Infarction Is Associated With Increased Risk of Cancer,” Journal of the American College of Cardiology 68 (2016): 265–271. 27417004 10.1016/j.jacc.2016.04.053 PMC4947209

[7] S. H. Armenian , C. Lacchetti , A. Barac , et al., “Prevention and Monitoring of Cardiac Dysfunction in Survivors of Adult Cancers: American Society of Clinical Oncology Clinical Practice Guideline,” Journal of Clinical Oncology 35 (2017): 893–911. 27918725 10.1200/JCO.2016.70.5400

[8] A. R. Lyon , T. López‐Fernández , L. S. Couch , et al., “2022 ESC Guidelines on Cardio‐oncology Developed in Collaboration With the European Hematology Association (EHA), the European Society for Therapeutic Radiology and Oncology (ESTRO) and the International Cardio‐Oncology Society (IC‐OS),” European Heart Journal 43 (2022): 4229–4361. 36017568 10.1093/eurheartj/ehac244

[9] K. M. Sturgeon , L. Deng , S. M. Bluethmann , et al., “A Population‐Based Study of Cardiovascular Disease Mortality Risk in US Cancer Patients,” European Heart Journal 40 (2019): 3889–3897. 31761945 10.1093/eurheartj/ehz766 PMC6925383

[10] W. C. Meijers , M. Maglione , S. J. Bakker , et al., “Heart Failure Stimulates Tumor Growth by Circulating Factors,” Circulation 138 (2018): 678–691. 29459363 10.1161/CIRCULATIONAHA.117.030816

[11] J. Moslehi , K. Fujiwara , and T. Guzik , “Cardio‐Oncology: A Novel Platform for Basic and Translational Cardiovascular Investigation Driven by Clinical Need,” Cardiovascular Research 115 (2019): 819–823. 30888396 10.1093/cvr/cvz048

[12] H. Strongman , S. Gadd , A. Matthews , et al., “Medium and Long‐Term Risks of Specific Cardiovascular Diseases in Survivors of 20 Adult Cancers: A Population‐Based Cohort Study Using Multiple Linked UK Electronic Health Records Databases,” Lancet 394 (2019): 1041–1054. 31443926 10.1016/S0140-6736(19)31674-5 PMC6857444

[13] Q. Li , G. Zhang , X. Li , et al., “Risk of Cardiovascular Disease Among Cancer Survivors: Systematic Review and Meta‐Analysis,” EClinicalMedicine 84 (2025): 103274. 40524799 10.1016/j.eclinm.2025.103274 PMC12169785

[14] W. E. Balch , R. I. Morimoto , A. Dillin , and J. W. Kelly , “Adapting Proteostasis for Disease Intervention,” Science 319 (2008): 916–919. 18276881 10.1126/science.1141448

[15] X. Wang and J. Robbins , “Proteasomal and Lysosomal Protein Degradation and Heart Disease,” Journal of Molecular and Cellular Cardiology 71 (2014): 16–24. 24239609 10.1016/j.yjmcc.2013.11.006 PMC4011941

[16] R. H. Henning and B. Brundel , “Proteostasis in Cardiac Health and Disease,” Nature Reviews Cardiology 14 (2017): 637–653. 28660894 10.1038/nrcardio.2017.89

[17] H. Bühringer , S. Doroudgar , X. Wang , N. Frey , and A. Y. Rangrez , “Editorial: Proteostasis in Cardiac Health and Disease,” Frontiers in Molecular Biosciences 11 (2024): 1433721. 38882607 10.3389/fmolb.2024.1433721 PMC11176937

[18] R. J. Deshaies , “Proteotoxic Crisis, the Ubiquitin‐Proteasome System, and Cancer Therapy,” BMC Biology 12 (2014): 94. 25385277 10.1186/s12915-014-0094-0 PMC4226866

[19] F. H. Schopf , M. M. Biebl , and J. Buchner , “The HSP90 Chaperone Machinery,” Nature Reviews Molecular Cell Biology 18 (2017): 345–360. 28429788 10.1038/nrm.2017.20

[20] E. E. Manasanch and R. Z. Orlowski , “Proteasome Inhibitors in Cancer Therapy,” Nature Reviews Clinical Oncology 14 (2017): 417–433. 10.1038/nrclinonc.2016.206 PMC5828026 28117417

[21] M. Taneike , O. Yamaguchi , A. Nakai , et al., “Inhibition of Autophagy in the Heart Induces Age‐Related Cardiomyopathy,” Autophagy 6 (2010): 600–606. 20431347 10.4161/auto.6.5.11947

[22] S. Lavandero , R. Troncoso , B. A. Rothermel , W. Martinet , J. Sadoshima , and J. A. Hill , “Cardiovascular Autophagy: Concepts, Controversies, and Perspectives,” Autophagy 9 (2013): 1455–1466. 23959233 10.4161/auto.25969

[23] J. Herrmann , “Adverse Cardiac Effects of Cancer Therapies: Cardiotoxicity and Arrhythmia,” Nature Reviews Cardiology 17 (2020): 474–502. 32231332 10.1038/s41569-020-0348-1 PMC8782611

[24] J. J. Moslehi and M. Deininger , “Tyrosine Kinase Inhibitor‐Associated Cardiovascular Toxicity in Chronic Myeloid Leukemia,” Journal of Clinical Oncology 33 (2015): 4210–4218. 26371140 10.1200/JCO.2015.62.4718 PMC4658454

[25] S. Sciarretta , Y. Maejima , D. Zablocki , and J. Sadoshima , “The Role of Autophagy in the Heart,” Annual Review of Physiology 80 (2018): 1–26. 10.1146/annurev-physiol-021317-121427 29068766

[26] A. Borlepawar , M. Neu , and Z. Ma , “Emerging Perspectives in Proteostasis: Bridging Mechanisms and Therapeutics for human Diseases,” Signal Transduction and Targeted Therapy 11 (2026): 224. 42259777 10.1038/s41392-026-02714-4 PMC13246805

[27] J. Labbadia and R. I. Morimoto , “The Biology of Proteostasis in Aging and Disease,” Annual Review of Biochemistry 84 (2015): 435–464. 10.1146/annurev-biochem-060614-033955 PMC4539002 25784053

[28] M. S. Hipp , P. Kasturi , and F. U. Hartl , “The Proteostasis Network and Its Decline in Ageing,” Nature Reviews Molecular Cell Biology 20 (2019): 421–435. 30733602 10.1038/s41580-019-0101-y

[29] S. Santaguida and A. Amon , “Short‐ and Long‐Term Effects of Chromosome Mis‐Segregation and Aneuploidy,” Nature Reviews Molecular Cell Biology 16 (2015): 473–485. 26204159 10.1038/nrm4025

[30] J. Luo , N. L. Solimini , and S. J. Elledge , “Principles of Cancer Therapy: Oncogene and Non‐Oncogene Addiction,” Cell 136 (2009): 823–837. 19269363 10.1016/j.cell.2009.02.024 PMC2894612

[31] J. J. Moslehi , “Cardiovascular Toxic Effects of Targeted Cancer Therapies,” New England Journal of Medicine 375 (2016): 1457–1467. 27732808 10.1056/NEJMra1100265

[32] B. Ky , P. Vejpongsa , E. T. Yeh , T. Force , and J. J. Moslehi , “Emerging Paradigms in Cardiomyopathies Associated With Cancer Therapies,” Circulation Research 113 (2013): 754–764. 23989717 10.1161/CIRCRESAHA.113.300218 PMC4046703

[33] J. Herrmann , E. H. Yang , C. A. Iliescu , et al., “Vascular Toxicities of Cancer Therapies: The Old and the New–An Evolving Avenue,” Circulation 133 (2016): 1272–1289. 27022039 10.1161/CIRCULATIONAHA.115.018347 PMC4817363

[34] E. White , “The Role for Autophagy in Cancer,” Journal of Clinical Investigation 125 (2015): 42–46. 25654549 10.1172/JCI73941 PMC4382247

[35] C. Hetz , K. Zhang , and R. J. Kaufman , “Mechanisms, Regulation and Functions of the Unfolded Protein Response,” Nature Reviews Molecular Cell Biology 21 (2020): 421–438. 32457508 10.1038/s41580-020-0250-z PMC8867924

[36] A. Hershko and A. Ciechanover , “The Ubiquitin System,” Annual Review of Biochemistry 67 (1998): 425–479. 10.1146/annurev.biochem.67.1.425 9759494

[37] A. Ciechanover , “The Ubiquitin‐Proteasome Pathway: On Protein Death and Cell Life,” Embo Journal 17 (1998): 7151–7160. 9857172 10.1093/emboj/17.24.7151 PMC1171061

[38] A. L. Goldberg , “Protein Degradation and Protection Against Misfolded or Damaged Proteins,” Nature 426 (2003): 895–899. 14685250 10.1038/nature02263

[39] D. Finley , “Recognition and Processing of Ubiquitin‐Protein Conjugates by the Proteasome,” Annual Review of Biochemistry 78 (2009): 477–513. 10.1146/annurev.biochem.78.081507.101607 PMC3431160 19489727

[40] M. Karin and Y. Ben‐Neriah , “Phosphorylation Meets Ubiquitination: The Control of NF‐[kappa]B Activity,” Annual Review of Immunology 18 (2000): 621–663. 10.1146/annurev.immunol.18.1.621 10837071

[41] M. H. Glickman and A. Ciechanover , “The Ubiquitin‐Proteasome Proteolytic Pathway: Destruction for the Sake of Construction,” Physiological Reviews 82 (2002): 373–428. 11917093 10.1152/physrev.00027.2001

[42] E. K. Schrader , K. G. Harstad , and A. Matouschek , “Targeting Proteins for Degradation,” Nature Chemical Biology 5 (2009): 815–822. 19841631 10.1038/nchembio.250 PMC4228941

[43] Y. C. Tang and A. Amon , “Gene Copy‐Number Alterations: A Cost‐Benefit Analysis,” Cell 152 (2013): 394–405. 23374337 10.1016/j.cell.2012.11.043 PMC3641674

[44] D. Popovic , D. Vucic , and I. Dikic , “Ubiquitination in Disease Pathogenesis and Treatment,” Nature Medicine 20 (2014): 1242–1253. 10.1038/nm.3739 25375928

[45] L. Bedford , J. Lowe , L. R. Dick , R. J. Mayer , and J. E. Brownell , “Ubiquitin‐Like Protein Conjugation and the Ubiquitin‐Proteasome System as Drug Targets,” Nature Reviews Drug Discovery 10 (2011): 29–46. 21151032 10.1038/nrd3321 PMC7097807

[46] J. Adams , “Potential for Proteasome Inhibition in the Treatment of Cancer,” Drug Discovery Today 8 (2003): 307–315. 12654543 10.1016/s1359-6446(03)02647-3

[47] A. Kisselev , W. A. van der Linden , and H. Overkleeft , “Proteasome Inhibitors: An Expanding Army Attacking a Unique Target,” Chemistry & Biology 19 (2012): 99–115. 22284358 10.1016/j.chembiol.2012.01.003 PMC3503453

[48] S. R. Powell , J. Herrmann , A. Lerman , C. Patterson , and X. Wang , “The Ubiquitin‐Proteasome System and Cardiovascular Disease,” Progress in Molecular Biology and Translational Science 109 (2012): 295–346. 22727426 10.1016/B978-0-12-397863-9.00009-2 PMC3743449

[49] M. S. Willis and C. Patterson , “Proteotoxicity and Cardiac Dysfunction,” New England Journal of Medicine 368 (2013): 1755. 23635068 10.1056/NEJMc1302511

[50] O. Tsukamoto , T. Minamino , and M. Kitakaze , “Functional Alterations of Cardiac Proteasomes Under Physiological and Pathological Conditions,” Cardiovascular Research 85 (2010): 339–346. 19684034 10.1093/cvr/cvp282

[51] J. Li , K. M. Horak , H. Su , A. Sanbe , J. Robbins , and X. Wang , “Enhancement of Proteasomal Function Protects Against Cardiac Proteinopathy and Ischemia/Reperfusion Injury in Mice,” Journal of Clinical Investigation 121 (2011): 3689–3700. 21841311 10.1172/JCI45709 PMC3163952

[52] J. M. Predmore , P. Wang , F. Davis , et al., “Ubiquitin Proteasome Dysfunction in human Hypertrophic and Dilated Cardiomyopathies,” Circulation 121 (2010): 997–1004. 20159828 10.1161/CIRCULATIONAHA.109.904557 PMC2857348

[53] C. Depre , Q. Wang , L. Yan , et al., “Activation of the Cardiac Proteasome During Pressure Overload Promotes Ventricular Hypertrophy,” Circulation 114 (2006): 1821–1828. 17043166 10.1161/CIRCULATIONAHA.106.637827

[54] C. Hu , Y. Tian , H. Xu , et al., “Inadequate Ubiquitination‐Proteasome Coupling Contributes to Myocardial Ischemia‐Reperfusion Injury,” Journal of Clinical Investigation 128 (2018): 5294–5306. 30204128 10.1172/JCI98287 PMC6264645

[55] D. Komander and M. Rape , “The Ubiquitin Code,” Annual Review of Biochemistry 81 (2012): 203–229. 10.1146/annurev-biochem-060310-170328 22524316

[56] F. Ikeda and I. Dikic , “Atypical Ubiquitin Chains: New Molecular Signals. ‘Protein Modifications: Beyond the Usual Suspects’ review Series,” Embo Reports 9 (2008): 536–542. 18516089 10.1038/embor.2008.93 PMC2427391

[57] Y. R. Lee , M. Chen , and P. P. Pandolfi , “The Functions and Regulation of the PTEN Tumour Suppressor: New Modes and Prospects,” Nature Reviews Molecular Cell Biology 19 (2018): 547–562. 29858604 10.1038/s41580-018-0015-0

[58] M. S. Song , L. Salmena , and P. P. Pandolfi , “The Functions and Regulation of the PTEN Tumour Suppressor,” Nature Reviews Molecular Cell Biology 13 (2012): 283–296. 22473468 10.1038/nrm3330

[59] V. Stambolic , A. Suzuki , J. L. de la Pompa , et al., “Negative Regulation of PKB/Akt‐Dependent Cell Survival by the Tumor Suppressor PTEN,” Cell 95 (1998): 29–39. 9778245 10.1016/s0092-8674(00)81780-8

[60] D. R. Alessi , S. R. James , C. Downes , et al., “Characterization of a 3‐phosphoinositide‐Dependent Protein Kinase Which Phosphorylates and Activates Protein Kinase Balpha,” Current Biology 7 (1997): 261–269. 9094314 10.1016/s0960-9822(06)00122-9

[61] W. L. Yang , C. Y. Wu , J. Wu , and H. K. Lin , “Regulation of Akt Signaling Activation by Ubiquitination,” Cell Cycle 9 (2010): 487–497. 20081374 10.4161/cc.9.3.10508 PMC3077544

[62] C. Chan , U. Jo , and A. Kohrman , “Posttranslational Regulation of Akt in human Cancer,” Cell Bioscience 4 (2014): 59. 25309720 10.1186/2045-3701-4-59 PMC4192732

[63] G. Y. Oudit , Z. Kassiri , J. Zhou , et al., “Loss of PTEN Attenuates the Development of Pathological Hypertrophy and Heart Failure in Response to Biomechanical Stress,” Cardiovascular Research 78 (2008): 505–514. 18281373 10.1093/cvr/cvn041

[64] M. A. Crackower , G. Y. Oudit , I. Kozieradzki , et al., “Regulation of Myocardial Contractility and Cell Size by Distinct PI3K‐PTEN Signaling Pathways,” Cell 110 (2002): 737–749. 12297047 10.1016/s0092-8674(02)00969-8

[65] M. Karin , “NF‐kappaB as a Critical Link Between Inflammation and Cancer,” Cold Spring Harbor Perspectives in Biology 1 (2009): a000141. 20066113 10.1101/cshperspect.a000141 PMC2773649

[66] M. S. Hayden and S. Ghosh , “NF‐kappaB in Immunobiology,” Cell Research 21 (2011): 223–244. 21243012 10.1038/cr.2011.13 PMC3193440

[67] I. E. Wertz and V. M. Dixit , “Regulation of Death Receptor Signaling by the Ubiquitin System,” Cell Death and Differentiation 17 (2010): 14–24. 19893571 10.1038/cdd.2009.168

[68] S. Frantz , J. Bauersachs , and G. Ertl , “Post‐Infarct Remodelling: Contribution of Wound Healing and Inflammation,” Cardiovascular Research 81 (2009): 474–481. 18977766 10.1093/cvr/cvn292 PMC2639128

[69] D. L. Mann , “Inflammatory Mediators and the Failing Heart: Past, Present, and the Foreseeable Future,” Circulation Research 91 (2002): 988–998. 12456484 10.1161/01.res.0000043825.01705.1b

[70] J. W. Gordon , J. A. Shaw , and L. A. Kirshenbaum , “Multiple Facets of NF‐kappaB in the Heart: To be or Not to NF‐kappaB,” Circulation Research 108 (2011): 1122–1132. 21527742 10.1161/CIRCRESAHA.110.226928

[71] N. Mizushima and M. Komatsu , “Autophagy: Renovation of Cells and Tissues,” Cell 147 (2011): 728–741. 22078875 10.1016/j.cell.2011.10.026

[72] B. Levine and G. Kroemer , “Autophagy in the Pathogenesis of Disease,” Cell 132 (2008): 27–42. 18191218 10.1016/j.cell.2007.12.018 PMC2696814

[73] L. Galluzzi , E. H. Baehrecke , A. Ballabio , et al., “Molecular Definitions of Autophagy and Related Processes,” Embo Journal 36 (2017): 1811–1836. 28596378 10.15252/embj.201796697 PMC5494474

[74] D. J. Klionsky and S. D. Emr , “Autophagy as a Regulated Pathway of Cellular Degradation,” Science 290 (2000): 1717–1721. 11099404 10.1126/science.290.5497.1717 PMC2732363

[75] R. Mathew , V. Karantza‐Wadsworth , and E. White , “Role of Autophagy in Cancer,” Nature Reviews Cancer 7 (2007): 961–967. 17972889 10.1038/nrc2254 PMC2866167

[76] A. Takamura , M. Komatsu , T. Hara , et al., “Autophagy‐Deficient Mice Develop Multiple Liver Tumors,” Genes & Development 25 (2011): 795–800. 21498569 10.1101/gad.2016211 PMC3078705

[77] A. C. Kimmelman and E. White , “Autophagy and Tumor Metabolism,” Cell Metabolism 25 (2017): 1037–1043. 28467923 10.1016/j.cmet.2017.04.004 PMC5604466

[78] J. Y. Guo , H. Chen , R. Mathew , et al., “Activated Ras Requires Autophagy to Maintain Oxidative Metabolism and Tumorigenesis,” Genes & Development 25 (2011): 460–470. 21317241 10.1101/gad.2016311 PMC3049287

[79] R. Amaravadi , A. C. Kimmelman , and E. White , “Recent Insights Into the Function of Autophagy in Cancer,” Genes & Development 30 (2016): 1913–1930. 27664235 10.1101/gad.287524.116 PMC5066235

[80] C. G. Towers and A. Thorburn , “Therapeutic Targeting of Autophagy,” EBioMedicine 14 (2016): 15–23. 28029600 10.1016/j.ebiom.2016.10.034 PMC5161418

[81] A. Nakai , O. Yamaguchi , T. Takeda , et al., “The Role of Autophagy in Cardiomyocytes in the Basal state and in Response to Hemodynamic Stress,” Nature Medicine 13 (2007): 619–624. 10.1038/nm1574 17450150

[82] A. Shirakabe , P. Zhai , Y. Ikeda , et al., “Drp1‐Dependent Mitochondrial Autophagy Plays a Protective Role Against Pressure Overload‐Induced Mitochondrial Dysfunction and Heart Failure,” Circulation 133 (2016): 1249–1263. 26915633 10.1161/CIRCULATIONAHA.115.020502 PMC4811679

[83] M. S. Bhuiyan , J. S. Pattison , H. Osinska , et al., “Enhanced Autophagy Ameliorates Cardiac Proteinopathy,” Journal of Clinical Investigation 123 (2013): 5284–5297. 24177425 10.1172/JCI70877 PMC3859422

[84] Y. Matsui , H. Takagi , X. Qu , et al., “Distinct Roles of Autophagy in the Heart During Ischemia and Reperfusion: Roles of AMP‐Activated Protein Kinase and Beclin 1 in Mediating Autophagy,” Circulation Research 100 (2007): 914–922. 17332429 10.1161/01.RES.0000261924.76669.36

[85] R. K. Amaravadi , D. Yu , J. J. Lum , et al., “Autophagy Inhibition Enhances Therapy‐Induced Apoptosis in a Myc‐Induced Model of Lymphoma,” Journal of Clinical Investigation 117 (2007): 326–336. 17235397 10.1172/JCI28833 PMC1765515

[86] V. W. Rebecca and R. K. Amaravadi , “Emerging Strategies to Effectively Target Autophagy in Cancer,” Oncogene 35 (2016): 1–11. 25893285 10.1038/onc.2015.99 PMC4838040

[87] J. Sadoshima , “The Role of Autophagy During Ischemia/Reperfusion,” Autophagy 4 (2008): 402–403. 18367869 10.4161/auto.5924

[88] I. Dikic , “Proteasomal and Autophagic Degradation Systems,” Annual Review of Biochemistry 86 (2017): 193–224. 10.1146/annurev-biochem-061516-044908 28460188

[89] A. M. Choi , S. W. Ryter , and B. Levine , “Autophagy in human Health and Disease,” New England Journal of Medicine 368 (2013): 651–662. 23406030 10.1056/NEJMra1205406

[90] D. A. Gewirtz , “The Four Faces of Autophagy: Implications for Cancer Therapy,” Cancer Research 74 (2014): 647–651. 24459182 10.1158/0008-5472.CAN-13-2966

[91] G. Filomeni , D. De Zio , and F. Cecconi , “Oxidative Stress and Autophagy: The Clash Between Damage and Metabolic Needs,” Cell Death and Differentiation 22 (2015): 377–388. 25257172 10.1038/cdd.2014.150 PMC4326572

[92] S. Sciarretta , M. Forte , G. Frati , and J. Sadoshima , “New Insights Into the Role of mTOR Signaling in the Cardiovascular System,” Circulation Research 122 (2018): 489–505. 29420210 10.1161/CIRCRESAHA.117.311147 PMC6398933

[93] L. Galluzzi , J. M. Bravo‐San Pedro , B. Levine , D. R. Green , and G. Kroemer , “Pharmacological Modulation of Autophagy: Therapeutic Potential and Persisting Obstacles,” Nature Reviews Drug Discovery 16 (2017): 487–511. 28529316 10.1038/nrd.2017.22 PMC5713640

[94] E. White , J. M. Mehnert , and C. S. A. Chan , “Metabolism, and Cancer,” Clinical Cancer Research 21 (2015): 5037–5046. 26567363 10.1158/1078-0432.CCR-15-0490 PMC4646728

[95] D. Ron and P. Walter , “Signal Integration in the Endoplasmic Reticulum Unfolded Protein Response,” Nature Reviews Molecular Cell Biology 8 (2007): 519–529. 17565364 10.1038/nrm2199

[96] C. Hetz , “The Unfolded Protein Response: Controlling Cell Fate Decisions Under ER Stress and Beyond,” Nature Reviews Molecular Cell Biology 13 (2012): 89–102. 22251901 10.1038/nrm3270

[97] S. Wang and R. J. Kaufman , “The Impact of the Unfolded Protein Response on human Disease,” Journal of Cell Biology 197 (2012): 857–867. 22733998 10.1083/jcb.201110131 PMC3384412

[98] P. Walter and D. Ron , “The Unfolded Protein Response: From Stress Pathway to Homeostatic Regulation,” Science 334 (2011): 1081–1086. 22116877 10.1126/science.1209038

[99] C. Hetz and F. R. Papa , “The Unfolded Protein Response and Cell Fate Control,” Molecular Cell 69 (2018): 169–181. 29107536 10.1016/j.molcel.2017.06.017

[100] B. M. Gardner , D. Pincus , K. Gotthardt , C. M. Gallagher , and P. Walter , “Endoplasmic Reticulum Stress Sensing in the Unfolded Protein Response,” Cold Spring Harbor Perspectives in Biology 5 (2013): a013169. 23388626 10.1101/cshperspect.a013169 PMC3578356

[101] J. R. Cubillos‐Ruiz , S. E. Bettigole , and L. H. Glimcher , “Molecular Pathways: Immunosuppressive Roles of IRE1alpha‐XBP1 Signaling in Dendritic Cells of the Tumor Microenvironment,” Clinical Cancer Research 22 (2016): 2121–2126. 26979393 10.1158/1078-0432.CCR-15-1570 PMC4854763

[102] H. P. Harding , Y. Zhang , and D. Ron , “Protein Translation and Folding Are Coupled by an Endoplasmic‐Reticulum‐Resident Kinase,” Nature 397 (1999): 271–274. 9930704 10.1038/16729

[103] K. Pakos‐Zebrucka , I. Koryga , K. Mnich , M. Ljujic , A. Samali , and A. M. Gorman , “The Integrated Stress Response,” Embo Reports 17 (2016): 1374–1395. 27629041 10.15252/embr.201642195 PMC5048378

[104] A. H. Lee , N. N. Iwakoshi , and L. H. Glimcher , “XBP‐1 Regulates a Subset of Endoplasmic Reticulum Resident Chaperone Genes in the Unfolded Protein Response,” Molecular and Cellular Biology 23 (2003): 7448–7459. 14559994 10.1128/MCB.23.21.7448-7459.2003 PMC207643

[105] H. Yoshida , T. Okada , and K. Haze , “ATF6 activated by Proteolysis Binds in the Presence of NF‐Y (CBF) Directly to the Cis‐Acting Element Responsible for the Mammalian Unfolded Protein Response,” Molecular and Cellular Biology 20 (2000): 6755–6767. 10958673 10.1128/mcb.20.18.6755-6767.2000 PMC86199

[106] H. L. Smith and G. R. Mallucci , “The Unfolded Protein Response: Mechanisms and Therapy of Neurodegeneration,” Brain 139 (2016): 2113–2121. 27190028 10.1093/brain/aww101 PMC4958893

[107] I. Tabas and D. Ron , “Integrating the Mechanisms of Apoptosis Induced by Endoplasmic Reticulum Stress,” Nature Cell Biology 13 (2011): 184–190. 21364565 10.1038/ncb0311-184 PMC3107571

[108] C. Hetz and S. Saxena , “ER Stress and the Unfolded Protein Response in Neurodegeneration,” Nature Reviews Neurology 13 (2017): 477–491. 28731040 10.1038/nrneurol.2017.99

[109] F. Urano , X. Wang , and A. Bertolotti , “Coupling of Stress in the ER to Activation of JNK Protein Kinases by Transmembrane Protein Kinase IRE1,” Science 287 (2000): 664–666. 10650002 10.1126/science.287.5453.664

[110] R. V. Rao , H. M. Ellerby , and D. E. Bredesen , “Coupling Endoplasmic Reticulum Stress to the Cell Death Program,” Cell Death and Differentiation 11 (2004): 372–380. 14765132 10.1038/sj.cdd.4401378

[111] J. Cubillos‐Ruiz , P. Silberman , M. Rutkowski , et al., “ER Stress Sensor XBP1 Controls Anti‐Tumor Immunity by Disrupting Dendritic Cell Homeostasis,” Cell 161 (2015): 1527–1538. 26073941 10.1016/j.cell.2015.05.025 PMC4580135

[112] T. Avril , E. Vauléon , and E. Chevet , “Endoplasmic Reticulum Stress Signaling and Chemotherapy Resistance in Solid Cancers,” Oncogenesis 6 (2017): e373. 28846078 10.1038/oncsis.2017.72 PMC5608920

[113] N. R. Mahadevan , J. Rodvold , H. Sepulveda , S. Rossi , A. F. Drew , and M. Zanetti , “Transmission of Endoplasmic Reticulum Stress and Pro‐Inflammation From Tumor Cells to Myeloid Cells,” Proceedings of the National Academy of Sciences of the United States of America 108 (2011): 6561–6566. 21464300 10.1073/pnas.1008942108 PMC3081038

[114] M. Bi , C. Naczki , M. Koritzinsky , et al., “ER Stress‐Regulated Translation Increases Tolerance to Extreme Hypoxia and Promotes Tumor Growth,” Embo Journal 24 (2005): 3470–3481. 16148948 10.1038/sj.emboj.7600777 PMC1276162

[115] M. Wang and R. J. Kaufman , “Protein Misfolding in the Endoplasmic Reticulum as a Conduit to human Disease,” Nature 529 (2016): 326–335. 26791723 10.1038/nature17041

[116] B. Luo and A. S. Lee , “The Critical Roles of Endoplasmic Reticulum Chaperones and Unfolded Protein Response in Tumorigenesis and Anticancer Therapies,” Oncogene 32 (2013): 805–818. 22508478 10.1038/onc.2012.130 PMC3819728

[117] K. M. Rouschop , L. J. Dubois , T. G. Keulers , et al., “PERK/eIF2alpha Signaling Protects Therapy Resistant Hypoxic Cells Through Induction of Glutathione Synthesis and Protection Against ROS,” Proceedings of the National Academy of Sciences of the United States of America 110 (2013): 4622–4627. 23471998 10.1073/pnas.1210633110 PMC3607059

[118] Y. Adachi , K. Yamamoto , T. Okada , H. Yoshida , A. Harada , and K. Mori , “ATF6 is a Transcription Factor Specializing in the Regulation of Quality Control Proteins in the Endoplasmic Reticulum,” Cell Structure and Function 33 (2008): 75–89. 18360008 10.1247/csf.07044

[119] R. L. Wiseman , J. S. Mesgarzadeh , and L. M. Hendershot , “Reshaping Endoplasmic Reticulum Quality Control Through the Unfolded Protein Response,” Molecular Cell 82 (2022): 1477–1491. 35452616 10.1016/j.molcel.2022.03.025 PMC9038009

[120] C. Salvagno , J. K. Mandula , P. C. Rodriguez , and J. R. Cubillos‐Ruiz , “Decoding Endoplasmic Reticulum Stress Signals in Cancer Cells and Antitumor Immunity,” Trends in Cancer 8 (2022): 930–943. 35817701 10.1016/j.trecan.2022.06.006 PMC9588488

[121] S. Marfatiya , F. Mubariz , A. Pal , et al., “Targeting the Unfolded Protein Response in Cancer: Exploiting Endoplasmic Reticulum Stress for Therapeutic Intervention,” Biochemical Pharmacology 242 (2025): 117221.40789371 10.1016/j.bcp.2025.117221

[122] S. Su , L. Wu , C. Huang , et al., “ATF6 activation Promotes Tumorigenesis and Drug Resistance in Diffuse Large B‐Cell Lymphoma (DLBCL) by Regulating the mTOR/S6K Signaling Pathway,” Discover Oncology 16 (2025): 499. 40205285 10.1007/s12672-025-02264-1 PMC11982007

[123] B. Unal and F. Saatcioglu , “Targeting the Unfolded Protein Response for Cancer Therapy: Mitigating Tumor Adaptation and Immune Suppression,” Biomarker Research 13 (2025): 156. 41372991 10.1186/s40364-025-00813-y PMC12696942

[124] S. Doroudgar , D. J. Thuerauf , M. C. Marcinko , P. J. Belmont , and C. C. Glembotski , “Ischemia Activates the ATF6 Branch of the Endoplasmic Reticulum Stress Response,” Journal of Biological Chemistry 284 (2009): 29735–29745. 19622751 10.1074/jbc.M109.018036 PMC2785605

[125] J. J. Martindale , R. Fernandez , D. Thuerauf , et al., “Endoplasmic Reticulum Stress Gene Induction and Protection From Ischemia/Reperfusion Injury in the Hearts of Transgenic Mice With a Tamoxifen‐Regulated Form of ATF6,” Circulation Research 98 (2006): 1186–1193. 16601230 10.1161/01.RES.0000220643.65941.8d

[126] D. J. Thuerauf , M. Marcinko , N. Gude , M. Rubio , M. A. Sussman , and C. C. Glembotski , “Activation of the Unfolded Protein Response in Infarcted Mouse Heart and Hypoxic Cultured Cardiac Myocytes,” Circulation Research 99 (2006): 275–282. 16794188 10.1161/01.RES.0000233317.70421.03

[127] S. Wang , P. Binder , Q. Fang , et al., “Endoplasmic Reticulum Stress in the Heart: Insights Into Mechanisms and Drug Targets,” British Journal of Pharmacology 175 (2018): 1293–1304. 28548229 10.1111/bph.13888 PMC5867005

[128] T. Minamino , I. Komuro , and M. Kitakaze , “Endoplasmic Reticulum Stress as a Therapeutic Target in Cardiovascular Disease,” Circulation Research 107 (2010): 1071–1082. 21030724 10.1161/CIRCRESAHA.110.227819

[129] M. S. Shah and M. Brownlee , “Molecular and Cellular Mechanisms of Cardiovascular Disorders in Diabetes,” Circulation Research 118 (2016): 1808–1829. 27230643 10.1161/CIRCRESAHA.116.306923 PMC4888901

[130] J. Groenendyk , L. B. Agellon , and M. Michalak , “Coping With Endoplasmic Reticulum Stress in the Cardiovascular System,” Annual Review of Physiology 75 (2013): 49–67. 10.1146/annurev-physiol-030212-183707 23020580

[131] C. Hetz , E. Chevet , and H. P. Harding , “Targeting the Unfolded Protein Response in Disease,” Nature Reviews Drug Discovery 12 (2013): 703–719. 23989796 10.1038/nrd3976

[132] J. R. Cubillos‐Ruiz , S. E. Bettigole , and L. H. Glimcher , “Tumorigenic and Immunosuppressive Effects of Endoplasmic Reticulum Stress in Cancer,” Cell 168 (2017): 692–706. 28187289 10.1016/j.cell.2016.12.004 PMC5333759

[133] F. U. Hartl , A. Bracher , and M. Hayer‐Hartl , “Molecular Chaperones in Protein Folding and Proteostasis,” Nature 475 (2011): 324–332. 21776078 10.1038/nature10317

[134] Y. E. Kim , M. S. Hipp , A. Bracher , M. Hayer‐Hartl , and F. Ulrich Hartl , “Molecular Chaperone Functions in Protein Folding and Proteostasis,” Annual Review of Biochemistry 82 (2013): 323–355. 10.1146/annurev-biochem-060208-092442 23746257

[135] M. Taipale , D. F. Jarosz , and S. Lindquist , “HSP90 at the Hub of Protein Homeostasis: Emerging Mechanistic Insights,” Nature Reviews Molecular Cell Biology 11 (2010): 515–528. 20531426 10.1038/nrm2918

[136] O. Genest , S. Wickner , and S. M. Doyle , “Hsp90 and Hsp70 Chaperones: Collaborators in Protein Remodeling,” Journal of Biological Chemistry 294 (2019): 2109–2120. 30401745 10.1074/jbc.REV118.002806 PMC6369297

[137] N. Kettern , M. Dreiseidler , R. Tawo , and J. Höhfeld , “Chaperone‐Assisted Degradation: Multiple Paths to Destruction,” Biological Chemistry 391 (2010): 481–489. 20302520 10.1515/BC.2010.058

[138] V. Arndt , C. Rogon , and J. Höhfeld , “To be, or Not to be–molecular Chaperones in Protein Degradation,” Cellular and Molecular Life Sciences 64 (2007): 2525–2541. 17565442 10.1007/s00018-007-7188-6 PMC11136327

[139] L. Whitesell and S. L. Lindquist , “HSP90 and the Chaperoning of Cancer,” Nature Reviews Cancer 5 (2005): 761–772. 16175177 10.1038/nrc1716

[140] H. H. Kampinga and E. A. Craig , “The HSP70 Chaperone Machinery: J Proteins as Drivers of Functional Specificity,” Nature Reviews Molecular Cell Biology 11 (2010): 579–592. 20651708 10.1038/nrm2941 PMC3003299

[141] M. Rohde , M. Daugaard , M. H. Jensen , K. Helin , J. Nylandsted , and M. Jäättelä , “Members of the Heat‐Shock Protein 70 family Promote Cancer Cell Growth by Distinct Mechanisms,” Genes & Development 19 (2005): 570–582. 15741319 10.1101/gad.305405 PMC551577

[142] D. Li , N. D. Marchenko , R. Schulz , et al., “Functional Inactivation of Endogenous MDM2 and CHIP by HSP90 Causes Aberrant Stabilization of Mutant p53 in human Cancer Cells,” Molecular Cancer Research 9 (2011): 577–588. 21478269 10.1158/1541-7786.MCR-10-0534 PMC3097033

[143] P. A. Muller and K. H. Vousden , “Mutant p53 in Cancer: New Functions and Therapeutic Opportunities,” Cancer Cell 25 (2014): 304–317. 24651012 10.1016/j.ccr.2014.01.021 PMC3970583

[144] E. M. Alexandrova , A. R. Yallowitz , and D. Li , “Improving Survival by Exploiting Tumour Dependence on Stabilized Mutant p53 for Treatment,” Nature 523 (2015): 352–356. 26009011 10.1038/nature14430 PMC4506213

[145] M. S. Martinho , D. J. Nancarrow , T. S. Lawrence , D. G. Beer , and D. Ray , “Chaperones and Ubiquitin Ligases Balance Mutant p53 Protein Stability in Esophageal and Other Digestive Cancers,” Cellular and Molecular Gastroenterology and Hepatology 11 (2021): 449–464. 33130332 10.1016/j.jcmgh.2020.10.012 PMC7788241

[146] P. Muller , R. Hrstka , D. Coomber , D. P. Lane , and B. Vojtesek , “Chaperone‐Dependent Stabilization and Degradation of p53 Mutants,” Oncogene 27 (2008): 3371–3383. 18223694 10.1038/sj.onc.1211010

[147] C. Sõti , E. Nagy , Z. Giricz , L. Vígh , P. Csermely , and P. Ferdinandy , “Heat Shock Proteins as Emerging Therapeutic Targets,” British Journal of Pharmacology 146 (2005): 769–780. 16170327 10.1038/sj.bjp.0706396 PMC1751210

[148] D. S. Latchman , “Heat Shock Proteins and Cardiac Protection,” Cardiovascular Research 51 (2001): 637–646. 11530097 10.1016/s0008-6363(01)00354-6

[149] A. A. Knowlton , S. Kapadia , G. Torre‐Amione , et al., “Differential Expression of Heat Shock Proteins in Normal and Failing human Hearts,” Journal of Molecular and Cellular Cardiology 30 (1998): 811–818. 9602430 10.1006/jmcc.1998.0646

[150] M. S. Willis and C. Patterson , “Hold Me Tight: Role of the Heat Shock Protein family of Chaperones in Cardiac Disease,” Circulation 122 (2010): 1740–1751. 20975010 10.1161/CIRCULATIONAHA.110.942250 PMC2976481

[151] S. Der Sarkissian , H. Aceros , P. M. Williams , C. Scalabrini , M. Borie , and N. Noiseux , “Heat Shock Protein 90 Inhibition and Multi‐Target Approach to Maximize Cardioprotection in Ischaemic Injury,” British Journal of Pharmacology 177 (2020): 3378–3388. 32335899 10.1111/bph.15075 PMC7348083

[152] N. Golenhofen , M. D. Perng , R. A. Quinlan , and D. Drenckhahn , “Comparison of the Small Heat Shock Proteins alphaB‐Crystallin, MKBP, HSP25, HSP20, and cvHSP in Heart and Skeletal Muscle,” Histochemistry and Cell Biology 122 (2004): 415–425. 15480735 10.1007/s00418-004-0711-z

[153] J. Trepel , M. Mollapour , G. Giaccone , and L. Neckers , “Targeting the Dynamic HSP90 Complex in Cancer,” Nature Reviews Cancer 10 (2010): 537–549. 20651736 10.1038/nrc2887 PMC6778733

[154] K. Jhaveri , T. Taldone , S. Modi , and G. Chiosis , “Advances in the Clinical Development of Heat Shock Protein 90 (Hsp90) Inhibitors in Cancers,” Biochimica Et Biophysica Acta 1823 (2012): 742–755. 22062686 10.1016/j.bbamcr.2011.10.008 PMC3288123

[155] X. Qian , M. Yao , J. Xu , N. Dong , and S. Chen , “From Cancer Therapy to Cardiac Safety: The Role of Proteostasis in Drug‐Induced Cardiotoxicity,” Frontiers in Pharmacology 15 (2024): 1472387. 39611175 10.3389/fphar.2024.1472387 PMC11602306

[156] Y. Zheng , S. Huang , B. Xie , et al., “Cardiovascular Toxicity of Proteasome Inhibitors in Multiple Myeloma Therapy,” Current Problems in Cardiology 48 (2023): 101536. 36481392 10.1016/j.cpcardiol.2022.101536

[157] P. G. Richardson , P. Sonneveld , M. W. Schuster , et al., “Bortezomib or High‐Dose Dexamethasone for Relapsed Multiple Myeloma,” New England Journal of Medicine 352 (2005): 2487–2498. 15958804 10.1056/NEJMoa043445

[158] P. G. Richardson , P. Sonneveld , M. Schuster , et al., “Extended Follow‐up of a Phase 3 Trial in Relapsed Multiple Myeloma: Final Time‐to‐event Results of the APEX Trial,” Blood 110 (2007): 3557–3560. 17690257 10.1182/blood-2006-08-036947

[159] A. J. Waxman , S. Clasen , W. Hwang , et al., “Carfilzomib‐Associated Cardiovascular Adverse Events: A Systematic Review and Meta‐Analysis,” JAMA Oncology 4 (2018): e174519. 29285538 10.1001/jamaoncol.2017.4519 PMC5885859

[160] Y. Xiao , J. Yin , J. Wei , and Z. Shang , “Incidence and Risk of Cardiotoxicity Associated With bortezomib in the Treatment of Cancer: A Systematic Review and Meta‐Analysis,” PLoS ONE 9 (2014): e87671. 24489948 10.1371/journal.pone.0087671 PMC3906186

[161] A. Chari , A. K. Stewart , S. D. Russell , et al., “Analysis of carfilzomib Cardiovascular Safety Profile Across Relapsed and/or Refractory Multiple Myeloma Clinical Trials,” Blood Advances 2 (2018): 1633–1644. 29991494 10.1182/bloodadvances.2017015545 PMC6039655

[162] M. A. Dimopoulos , P. Moreau , A. Palumbo , et al., “Carfilzomib and Dexamethasone versus Bortezomib and Dexamethasone for Patients With Relapsed or Refractory Multiple Myeloma (ENDEAVOR): A Randomised, Phase 3, Open‐Label, Multicentre Study,” The Lancet Oncology 17 (2016): 27–38. 26671818 10.1016/S1470-2045(15)00464-7

[163] M. A. Dimopoulos , H. Goldschmidt , R. Niesvizky , et al., “Carfilzomib or Bortezomib in Relapsed or Refractory Multiple Myeloma (ENDEAVOR): An Interim Overall Survival Analysis of an Open‐Label, Randomised, Phase 3 Trial,” The Lancet Oncology 18 (2017): 1327–1337. 28843768 10.1016/S1470-2045(17)30578-8

[164] R. Z. Orlowski , P. Moreau , R. Niesvizky , et al., “Carfilzomib‐Dexamethasone versus Bortezomib‐Dexamethasone in Relapsed or Refractory Multiple Myeloma: Updated Overall Survival, Safety, and Subgroups,” Clinical Lymphoma, Myeloma & Leukemia 19 (2019): 522–530 e521. 10.1016/j.clml.2019.04.018 31160237

[165] G. Georgiopoulos , N. Makris , A. Laina , et al., “Cardiovascular Toxicity of Proteasome Inhibitors: Underlying Mechanisms and Management Strategies: JACC: CardioOncology State‐of‐the‐Art Review,” JACC: CardioOncology 5 (2023): 1–21. 36875897 10.1016/j.jaccao.2022.12.005 PMC9982226

[166] P. Wu , O. Oren , M. A. Gertz , and E. H. Yang , “Proteasome Inhibitor‐Related Cardiotoxicity: Mechanisms, Diagnosis, and Management,” Current Oncology Reports 22 (2020): 66. 32514632 10.1007/s11912-020-00931-w

[167] M. Wang and R. J. Kaufman , “The Impact of the Endoplasmic Reticulum Protein‐Folding Environment on Cancer Development,” Nature Reviews Cancer 14 (2014): 581–597. 25145482 10.1038/nrc3800

[168] Y. Kim , J. Suarez , and Y. Hu , “Deletion of the Inducible 70‐kDa Heat Shock Protein Genes in Mice Impairs Cardiac Contractile Function and Calcium Handling Associated With Hypertrophy,” Circulation 113 (2006): 2589–2597. 16735677 10.1161/CIRCULATIONAHA.105.598409

[169] J. Ren , Y. Bi , J. R. Sowers , C. Hetz , and Y. Zhang , “Endoplasmic Reticulum Stress and Unfolded Protein Response in Cardiovascular Diseases,” Nature Reviews Cardiology 18 (2021): 499–521. 33619348 10.1038/s41569-021-00511-w

[170] P. Pantziarka , “Repurposing Drugs in Oncology (ReDO)‐Mebendazole as an Anti‐Cancer Agent,” Ecancermedicalscience 8 (2014): 443. 25075217 10.3332/ecancer.2014.443 PMC4096024

[171] T. T. Ashburn and K. B. Thor , “Drug Repositioning: Identifying and Developing New Uses for Existing Drugs,” Nature Reviews Drug Discovery 3 (2004): 673–683. 15286734 10.1038/nrd1468

[172] T. I. Barron , R. M. Connolly , L. Sharp , K. Bennett , and K. Visvanathan , “Beta Blockers and Breast Cancer Mortality: A Population‐Based Study,” Journal of Clinical Oncology 29 (2011): 2635–2644. 21632503 10.1200/JCO.2010.33.5422

[173] W. K. Childers , C. S. Hollenbeak , and P. Cheriyath , “beta‐Blockers Reduce Breast Cancer Recurrence and Breast Cancer Death: A Meta‐Analysis,” Clinical Breast Cancer 15 (2015): 426–431. 26516037 10.1016/j.clbc.2015.07.001

[174] M. Coelho , M. Moz , G. Correia , A. Teixeira , R. Medeiros , and L. Ribeiro , “Antiproliferative Effects of Beta‐Blockers on human Colorectal Cancer Cells,” Oncology Reports 33 (2015): 2513–2520. 25812650 10.3892/or.2015.3874

[175] S. F. Nielsen , B. G. Nordestgaard , and S. E. Bojesen , “Statin Use and Reduced Cancer‐Related Mortality,” New England Journal of Medicine 367 (2012): 1792–1802. 23134381 10.1056/NEJMoa1201735

[176] S. Zhong , X. Zhang , L. Chen , T. Ma , J. Tang , and J. Zhao , “Statin Use and Mortality in Cancer Patients: Systematic Review and Meta‐Analysis of Observational Studies,” Cancer Treatment Reviews 41 (2015): 554–567. 25890842 10.1016/j.ctrv.2015.04.005

[177] Y. Li , X. He , Y. Ding , H. Chen , and L. Sun , “Statin Uses and Mortality in Colorectal Cancer Patients: An Updated Systematic Review and Meta‐Analysis,” Cancer Medicine 8 (2019): 3305–3313. 31069997 10.1002/cam4.2151 PMC6558478

[178] I. Sipahi , S. M. Debanne , D. Y. Rowland , D. I. Simon , and J. C. Fang , “Angiotensin‐Receptor Blockade and Risk of Cancer: Meta‐Analysis of Randomised Controlled Trials,” The Lancet Oncology 11 (2010): 627–636. 20542468 10.1016/S1470-2045(10)70106-6 PMC4070221

[179] S. Bangalore , S. Kumar , S. E. Kjeldsen , et al., “Antihypertensive Drugs and Risk of Cancer: Network Meta‐Analyses and Trial Sequential Analyses of 324,168 Participants From Randomised Trials,” The Lancet Oncology 12 (2011): 65–82. 21123111 10.1016/S1470-2045(10)70260-6

[180] B. M. Hicks , K. B. Filion , H. Yin , L. Sakr , J. A. Udell , and L. Azoulay , “Angiotensin Converting Enzyme Inhibitors and Risk of Lung Cancer: Population Based Cohort Study,” Bmj 363 (2018): k4209. 30355745 10.1136/bmj.k4209 PMC6199558

[181] K. Regulska , M. Regulski , B. Karolak , M. Murias , and B. Stanisz , “Can Cardiovascular Drugs Support Cancer Treatment? The Rationale for Drug Repurposing,” Drug Discovery Today 24 (2019): 1059–1065. 30878563 10.1016/j.drudis.2019.03.010

[182] D. Soranna , L. Scotti , A. Zambon , et al., “Cancer Risk Associated With Use of Metformin and Sulfonylurea in Type 2 Diabetes: A Meta‐Analysis,” The Oncologist 17 (2012): 813–822. 22643536 10.1634/theoncologist.2011-0462 PMC3380880

[183] M. Y. Zamanian , M. Golmohammadi , A. Yumashev , et al., “Effects of Metformin on Cancers in Experimental and Clinical Studies: Focusing on Autophagy and AMPK/mTOR Signaling Pathways,” Cell Biochemistry and Function 42 (2024): e4071. 38863255 10.1002/cbf.4071

[184] D. M. Boudreau , O. Yu , and J. Johnson , “Statin Use and Cancer Risk: A Comprehensive Review,” Expert Opinion on Drug Safety 9 (2010): 603–621. 20377474 10.1517/14740331003662620 PMC2910322

[185] S. Safwat , R. A. Ishak , R. M. Hathout , and N. D. Mortada , “Statins Anticancer Targeted Delivery Systems: Re‐Purposing an Old Molecule,” Journal of Pharmacy and Pharmacology 69 (2017): 613–624. 28271498 10.1111/jphp.12707

[186] K. Regulska , B. Stanisz , and M. Regulski , “The Renin‐Angiotensin System as a Target of Novel Anticancer Therapy,” Current Pharmaceutical Design 19 (2013): 7103–7125. 23859547 10.2174/13816128113199990508

[187] S. W. Cole , A. S. Nagaraja , S. K. Lutgendorf , P. A. Green , and A. K. Sood , “Sympathetic Nervous System Regulation of the Tumour Microenvironment,” Nature Reviews Cancer 15 (2015): 563–572. 26299593 10.1038/nrc3978 PMC4828959

[188] B. W. Renz , R. Takahashi , T. Tanaka , et al., “beta2 Adrenergic‐Neurotrophin Feedforward Loop Promotes Pancreatic Cancer,” Cancer Cell 33 (2018): 75–90 e77. 29249692 10.1016/j.ccell.2017.11.007 PMC5760435

[189] N. F. Z. Schneider , C. Cerella , C. M. O. Simoes , and M. Diederich , “Anticancer and Immunogenic Properties of Cardiac Glycosides,” Molecules 22 (2017): 1932. 29117117 10.3390/molecules22111932 PMC6150164

[190] G. Curigliano , D. Cardinale , S. Dent , et al., “Cardiotoxicity of Anticancer Treatments: Epidemiology, Detection, and Management,” CA: A Cancer Journal for Clinicians 66 (2016): 309–325. 26919165 10.3322/caac.21341

[191] E. C. van Dalen , H. J. van der Pal , and L. C. Kremer , “Different Dosage Schedules for Reducing Cardiotoxicity in People With Cancer Receiving Anthracycline Chemotherapy,” Cochrane Database of Systematic Reviews 3 (2016): CD005008. 26938118 10.1002/14651858.CD005008.pub4 PMC6457744

[192] C. Lewinter , T. H. Nielsen , L. R. Edfors , et al., “A Systematic Review and Meta‐Analysis of Beta‐Blockers and Renin‐Angiotensin System Inhibitors for Preventing Left Ventricular Dysfunction due to Anthracyclines or Trastuzumab in Patients With Breast Cancer,” European Heart Journal 43 (2022): 2562–2569. 34951629 10.1093/eurheartj/ehab843

[193] K. Kalam and T. H. Marwick , “Role of Cardioprotective Therapy for Prevention of Cardiotoxicity With Chemotherapy: A Systematic Review and Meta‐Analysis,” European Journal of Cancer 49 (2013): 2900–2909. 23706982 10.1016/j.ejca.2013.04.030

[194] G. Batist , G. Ramakrishnan , C. S. Rao , et al., “Reduced Cardiotoxicity and Preserved Antitumor Efficacy of Liposome‐Encapsulated Doxorubicin and Cyclophosphamide Compared With Conventional Doxorubicin and Cyclophosphamide in a Randomized, Multicenter Trial of Metastatic Breast Cancer,” Journal of Clinical Oncology 19 (2001): 1444–1454. 11230490 10.1200/JCO.2001.19.5.1444

[195] T. Safra , “Cardiac Safety of Liposomal Anthracyclines,” The Oncologist 8, no. Suppl 2 (2003): 17–24. 13679592 10.1634/theoncologist.8-suppl_2-17

[196] J. C. Plana , M. Galderisi , A. Barac , et al., “Expert Consensus for Multimodality Imaging Evaluation of Adult Patients During and After Cancer Therapy: A Report From the American Society of Echocardiography and the European Association of Cardiovascular Imaging,” European Heart Journal ‐ Cardiovascular Imaging 15 (2014): 1063–1093. 25239940 10.1093/ehjci/jeu192 PMC4402366

[197] P. Thavendiranathan , F. Poulin , K. Lim , J. C. Plana , A. Woo , and T. H. Marwick , “Use of Myocardial Strain Imaging by Echocardiography for the Early Detection of Cardiotoxicity in Patients During and After Cancer Chemotherapy: A Systematic Review,” Journal of the American College of Cardiology 63 (2014): 2751–2768. 24703918 10.1016/j.jacc.2014.01.073

[198] M. S. Willis and C. Patterson , “Proteotoxicity and Cardiac Dysfunction–Alzheimer's Disease of the Heart?,” New England Journal of Medicine 368 (2013): 455–464. 23363499 10.1056/NEJMra1106180

[199] S. K. Calderwood and J. Gong , “Heat Shock Proteins Promote Cancer: It's a Protection Racket,” Trends in Biochemical Sciences 41 (2016): 311–323. 26874923 10.1016/j.tibs.2016.01.003 PMC4911230

[200] A. De Maio , “Extracellular Heat Shock Proteins, Cellular Export Vesicles, and the Stress Observation System: A Form of Communication During Injury, Infection, and Cell Damage. It Is Never Known How Far a Controversial Finding Will Go! Dedicated to Ferruccio Ritossa,” Cell Stress & Chaperones 16 (2011): 235–249. 20963644 10.1007/s12192-010-0236-4 PMC3077223

[201] A. G. Pockley and B. Henderson , “Extracellular Cell Stress (heat shock) Proteins‐Immune Responses and Disease: An Overview,” Philosophical Transactions of the Royal Society of London. Series B: Biological Sciences 373 (2018): 20160522. 29203707 10.1098/rstb.2016.0522 PMC5717522

[202] A. S. Lee , “GRP78 induction in Cancer: Therapeutic and Prognostic Implications,” Cancer Research 67 (2007): 3496–3499. 17440054 10.1158/0008-5472.CAN-07-0325

[203] C. Roller and D. Maddalo , “The Molecular Chaperone GRP78/BiP in the Development of Chemoresistance: Mechanism and Possible Treatment,” Frontiers in Pharmacology 4 (2013): 10. 23403503 10.3389/fphar.2013.00010 PMC3568707

[204] D. Dong , M. Ni , J. Li , et al., “Critical Role of the Stress Chaperone GRP78/BiP in Tumor Proliferation, Survival, and Tumor Angiogenesis in Transgene‐Induced Mammary Tumor Development,” Cancer Research 68 (2008): 498–505. 18199545 10.1158/0008-5472.CAN-07-2950

[205] S. B. Scruggs , K. Watson , A. I. Su , et al., “Harnessing the Heart of Big Data,” Circulation Research 116 (2015): 1115–1119. 25814682 10.1161/CIRCRESAHA.115.306013 PMC4721634

[206] R. A. de Boer , J. S. Hulot , C. G. Tocchetti , et al., “Common Mechanistic Pathways in Cancer and Heart Failure. A Scientific Roadmap on Behalf of the Translational Research Committee of the Heart Failure Association (HFA) of the European Society of Cardiology (ESC),” European Journal of Heart Failure 22 (2020): 2272–2289. 33094495 10.1002/ejhf.2029 PMC7894564

[207] S. A. Williams , M. Kivimaki , C. Langenberg , et al., “Plasma Protein Patterns as Comprehensive Indicators of Health,” Nature Medicine 25 (2019): 1851–1857. 10.1038/s41591-019-0665-2 PMC6922049 31792462

[208] D. Rasooly , C. Giambartolomei , G. M. Peloso , et al., “Large‐Scale Multi‐Omics Identifies Drug Targets for Heart Failure With Reduced and Preserved Ejection Fraction,” Nature Cardiovascular Research 4 (2025): 293–311. 10.1038/s44161-025-00609-1 PMC13214498 39915329

[209] S. Chakraborty , G. Sharma , S. Karmakar , and S. Banerjee , “Multi‐OMICS Approaches in Cancer Biology: New Era in Cancer Therapy,” Biochimica et Biophysica Acta ‐ Molecular Basis of Disease 1870 (2024): 167120. 38484941 10.1016/j.bbadis.2024.167120

[210] P. Efentakis , G. Kremastiotis , A. Varela , et al., “Molecular Mechanisms of Carfilzomib‐Induced Cardiotoxicity in Mice and the Emerging Cardioprotective Role of Metformin,” Blood 133 (2019): 710–723. 30482794 10.1182/blood-2018-06-858415

[211] P. Efentakis , G. Psarakou , A. Varela , et al., “Elucidating Carfilzomib's Induced Cardiotoxicity in an in Vivo Model of Aging: Prophylactic Potential of Metformin,” International Journal of Molecular Sciences 22 (2021): 10956. 34681615 10.3390/ijms222010956 PMC8537073

[212] S. Ljubojević‐Holzer , S. Kraler , N. Djalinac , et al., “Loss of Autophagy Protein ATG5 Impairs Cardiac Capacity in Mice and Humans Through Diminishing Mitochondrial Abundance and Disrupting Ca2+ Cycling,” Cardiovascular Research 118 (2022): 1492–1505. 33752242 10.1093/cvr/cvab112 PMC9074988

[213] H. Toko , H. Takahashi , Y. Kayama , et al., “ATF6 is Important Under both Pathological and Physiological States in the Heart,” Journal of Molecular and Cellular Cardiology 49 (2010): 113–120. 20380836 10.1016/j.yjmcc.2010.03.020

[214] J. Jin , E. A. Blackwood , K. Azizi , et al., “ATF6 Decreases Myocardial Ischemia/Reperfusion Damage and Links ER Stress and Oxidative Stress Signaling Pathways in the Heart,” Circulation Research 120 (2017): 862–875. 27932512 10.1161/CIRCRESAHA.116.310266 PMC5336510

[215] S. Liu , Y. Bi , T. Han , et al., “The E3 Ubiquitin Ligase MARCH2 Protects Against Myocardial Ischemia‐Reperfusion Injury Through Inhibiting Pyroptosis via Negative Regulation of PGAM5/MAVS/NLRP3 Axis,” Cell Discovery 10 (2024): 24. 38409220 10.1038/s41421-023-00622-3 PMC10897310

[216] A. M. Cyran and A. Zhitkovich , “Heat Shock Proteins and HSF1 in Cancer,” Frontiers in Oncology 12 (2022): 860320. 35311075 10.3389/fonc.2022.860320 PMC8924369

[217] P. Moreau , T. Masszi , N. Grzasko , et al., “Oral Ixazomib, Lenalidomide, and Dexamethasone for Multiple Myeloma,” New England Journal of Medicine 374 (2016): 1621–1634. 27119237 10.1056/NEJMoa1516282

[218] T. Facon , C. P. Venner , N. J. Bahlis , et al., “Oral Ixazomib, Lenalidomide, and Dexamethasone for Transplant‐Ineligible Patients With Newly Diagnosed Multiple Myeloma,” Blood 137 (2021): 3616–3628. 33763699 10.1182/blood.2020008787 PMC8462404

[219] M. A. Dimopoulos , F. Schjesvold , V. Doronin , et al., “Oral Ixazomib‐Dexamethasone vs Oral Pomalidomide‐Dexamethasone for Lenalidomide‐Refractory, Proteasome Inhibitor‐Exposed Multiple Myeloma: A Randomized Phase 2 Trial,” Blood Cancer Journal 12 (2022): 9. 35075109 10.1038/s41408-021-00593-2 PMC8786921

[220] R. Bishnoi , Z. Xie , and C. Shah , “Real‐World Experience of carfilzomib‐Associated Cardiovascular Adverse Events: SEER‐Medicare Data Set Analysis,” Cancer Medicine 10 (2021): 70–78. 33169938 10.1002/cam4.3568 PMC7826471

[221] R. Xu , Z. Ji , C. Xu , and J. Zhu , “The Clinical Value of Using Chloroquine or Hydroxychloroquine as Autophagy Inhibitors in the Treatment of Cancers: A Systematic Review and Meta‐Analysis,” Medicine (Baltimore) 97 (2018): e12912. 30431566 10.1097/MD.0000000000012912 PMC6257684

[222] C. Verbaanderd , H. Maes , M. B. Schaaf , et al., “Repurposing Drugs in Oncology (ReDO)‐Chloroquine and Hydroxychloroquine as Anti‐Cancer Agents,” Ecancermedicalscience 11 (2017): 781. 29225688 10.3332/ecancer.2017.781 PMC5718030

[223] C. Chatre , F. Roubille , H. Vernhet , C. Jorgensen , and Y. M. Pers , “Cardiac Complications Attributed to Chloroquine and Hydroxychloroquine: A Systematic Review of the Literature,” Drug Safety 41 (2018): 919–931. 29858838 10.1007/s40264-018-0689-4

[224] S. Rastogi , A. Joshi , N. Sato , et al., “An Update on the Status of HSP90 Inhibitors in Cancer Clinical Trials,” Cell Stress & Chaperones 29 (2024): 519–539. 38878853 10.1016/j.cstres.2024.05.005 PMC11260857

[225] M. E. Stokes , V. Calvo , S. Fujisawa , et al., “PERK Inhibition by HC‐5404 Sensitizes Renal Cell Carcinoma Tumor Models to Antiangiogenic Tyrosine Kinase Inhibitors,” Clinical Cancer Research 29 (2023): 4870–4882. 37733811 10.1158/1078-0432.CCR-23-1182 PMC10690095

[226] D. Petrylak , B. Garmezy , J. Shen , et al., “Phase I/II Study of Bavdegalutamide, a PROTAC Androgen Receptor (AR) Degrader in Metastatic Castration‐Resistant Prostate Cancer (mCRPC): Radiographic Progression‐Free Survival (rPFS) in Patients (pts) With AR Ligand‐Binding Domain (LBD) Mutations,” Annals of Oncology 34 (2023): S973–S974.

[227] E. P. Hamilton , C. Ma , M. De Laurentiis , et al., “VERITAC‐2: A Phase III Study of vepdegestrant, a PROTAC ER Degrader, versus Fulvestrant in ER+/HER2‐ advanced Breast Cancer,” Future Oncology 20 (2024): 2447–2455. 39072356 10.1080/14796694.2024.2377530 PMC11524203

[228] M. Campone , M. De Laurentiis , K. Jhaveri , et al., “Vepdegestrant, a PROTAC Estrogen Receptor Degrader, in Advanced Breast Cancer,” New England Journal of Medicine 393 (2025): 556–568. 40454645 10.1056/NEJMoa2505725

[229] S. J. Jacobus , S. V. Rajkumar , M. Weiss , et al., “Randomized Phase III Trial of Consolidation Therapy With Bortezomib‐Lenalidomide‐Dexamethasone (VRd) vs Bortezomib‐Dexamethasone (Vd) for Patients With Multiple Myeloma Who Have Completed a Dexamethasone Based Induction Regimen,” Blood Cancer Journal 6 (2016): e448. 27471864 10.1038/bcj.2016.55 PMC5030380

[230] R. Hájek , R. Bryce , S. Ro , B. Klencke , and H. Ludwig , “Design and Rationale of FOCUS (PX‐171‐011): A Randomized, Open‐Label, Phase 3 Study of carfilzomib versus Best Supportive Care Regimen in Patients With Relapsed and Refractory Multiple Myeloma (R/R MM),” BMC Cancer 12 (2012): 415. 22992303 10.1186/1471-2407-12-415 PMC3489882

[231] D. Daniely , E. Forouzan , T. M. Spektor , et al., “A Phase 1/2 Study of ixazomib in Place of Bortezomib or Carfilzomib in a Subsequent Line of Therapy for Patients With Multiple Myeloma Refractory to Their Last Bortezomib or Carfilzomib Combination Regimen,” Experimental Hematology 111 (2022): 79–86. 35417741 10.1016/j.exphem.2022.04.003

[232] S. Ramalingam , G. Goss , R. Rosell , et al., “A Randomized Phase II Study of ganetespib, a Heat Shock Protein 90 Inhibitor, in Combination With docetaxel in Second‐Line Therapy of Advanced Non‐Small Cell Lung Cancer (GALAXY‐1),” Annals of Oncology 26 (2015): 1741–1748. 25997818 10.1093/annonc/mdv220

[233] M. K. Thakur , L. K. Heilbrun , S. Sheng , et al., “A Phase II Trial of ganetespib, a Heat Shock Protein 90 (Hsp90) Inhibitor, in Patients With Docetaxel‐Pretreated Metastatic Castrate‐Resistant Prostate Cancer (CRPC)‐a Prostate Cancer Clinical Trials Consortium (PCCTC) Study,” Investigational New Drugs 34 (2016): 112–118. 26581400 10.1007/s10637-015-0307-6 PMC6820516

[234] L. Huang , Y. Wang , J. Bai , et al., “Blockade of HSP70 by VER‐155008 Synergistically Enhances Bortezomib‐Induced Cytotoxicity in Multiple Myeloma,” Cell Stress & Chaperones 25 (2020): 357–367. 32026316 10.1007/s12192-020-01078-0 PMC7058745

[235] A. I. P. Eugênio , V. L. Fook‐Alves , M. B. de Oliveira , et al., “Proteasome and Heat Shock Protein 70 (HSP70) Inhibitors as Therapeutic Alternative in Multiple Myeloma,” Oncotarget 8 (2017): 114698–114709. 29383113 10.18632/oncotarget.22815 PMC5777725

[236] Y. Li , X. Liu , L. Yu , et al., “Covalent LYTAC Enabled by DNA Aptamers for Immune Checkpoint Degradation Therapy,” Journal of the American Chemical Society 145, no. 45 (2023): 24506–24521. 10.1021/jacs.3c03899 37910771

[237] G. Ahn , S. M. Banik , C. L. Miller , N. M. Riley , J. R. Cochran , and C. R. Bertozzi , “LYTACs That Engage the Asialoglycoprotein Receptor for Targeted Protein Degradation,” Nature Chemical Biology 17 (2021): 937–946. 33767387 10.1038/s41589-021-00770-1 PMC8387313

[238] S. M. Banik , K. Pedram , S. Wisnovsky , G. Ahn , N. M. Riley , and C. R. Bertozzi , “Lysosome‐Targeting Chimaeras for Degradation of Extracellular Proteins,” Nature 584 (2020): 291–297. 32728216 10.1038/s41586-020-2545-9 PMC7727926

[239] J. J. McMurray , S. D. Solomon , S. E. Inzucchi , et al., “Dapagliflozin in Patients With Heart Failure and Reduced Ejection Fraction,” New England Journal of Medicine 381 (2019): 1995–2008. 31535829 10.1056/NEJMoa1911303

[240] C. V. Thorup , C. N. A. Jeppesen , T. H. Jensen , et al., “POLYamine Treatment in Elderly Patients With Coronary Artery Disease (POLYCAD): Study Protocol for a Danish Randomised, Double‐Blind, Placebo‐Controlled Trial of Spermidine Treatment versus Placebo,” Trials 26 (2025): 452. 41168834 10.1186/s13063-025-09176-z PMC12574056

[241] A. Gyongyosi , N. Csaki , and A. Peto , “BGP‐15 Protects Against Doxorubicin‐Induced Cell Toxicity via Enhanced Mitochondrial Function,” International Journal of Molecular Sciences 24 (2023): 5269. 36982341 10.3390/ijms24065269 PMC10049233

[242] B. Bernat , R. Erdelyi , L. Fazekas , et al., “Drug Candidate BGP‐15 Prevents Isoproterenol‐Induced Arrhythmias and Alters Heart Rate Variability (HRV) in Telemetry‐Implanted Rats,” Pharmaceuticals (Basel) 16 (2023): 359. 36986459 10.3390/ph16030359 PMC10056898

[243] D. Priksz , N. Lampe , A. Kovacs , et al., “Nicotinic‐Acid Derivative BGP‐15 Improves Diastolic Function in a Rabbit Model of Atherosclerotic Cardiomyopathy,” British Journal of Pharmacology 179 (2022): 2240–2258. 34811751 10.1111/bph.15749

[244] O. Horvath , K. Ordog , K. Bruszt , et al., “BGP‐15 Protects Against Heart Failure by Enhanced Mitochondrial Biogenesis and Decreased Fibrotic Remodelling in Spontaneously Hypertensive Rats,” Oxidative Medicine and Cellular Longevity 2021 (2021): 1250858. 33564362 10.1155/2021/1250858 PMC7867468

[245] G. Mingrone , A. Astarita , L. Airale , et al., “Effects of Carfilzomib Therapy on Left Ventricular Function in Multiple Myeloma Patients,” Frontiers in Cardiovascular Medicine 8 (2021): 645678. 33969010 10.3389/fcvm.2021.645678 PMC8096903

[246] R. Singh , D. Sperling , A. Delicce , et al., “Changes in Global Longitudinal Strain as a Predictor of Cardiotoxicity After Exposure to Carfilzomib,” American Journal of Cardiology 217 (2024): 29–30. 38432340 10.1016/j.amjcard.2024.02.029

[247] A. Chari and D. Hajje , “Case Series Discussion of Cardiac and Vascular Events Following Carfilzomib Treatment: Possible Mechanism, Screening, and Monitoring,” BMC Cancer 14 (2014): 915. 25471129 10.1186/1471-2407-14-915 PMC4289164

[248] G. Mingrone , A. Astarita , A. Colomba , et al., “Patients With Very High Risk of Cardiovascular Adverse Events During Carfilzomib Therapy: Prevention and Management of Events in a Single Center Experience,” Cancers (Basel) 15 (2023): 1149. 36831492 10.3390/cancers15041149 PMC9953901

[249] Y. Qi , Y. Wei , L. Li , et al., “Genetic Factors in the Pathogenesis of Cardio‐Oncology,” Journal of Translational Medicine 22 (2024): 739. 39103883 10.1186/s12967-024-05537-5 PMC11301970

[250] Y. Kim , J. G. Seidman , and C. E. Seidman , “Genetics of Cancer Therapy‐Associated Cardiotoxicity,” Journal of Molecular and Cellular Cardiology 167 (2022): 85–91. 35358500 10.1016/j.yjmcc.2022.03.010 PMC9107514

[251] L. Martínez‐Campelo , A. Blanco‐Verea , T. López‐Fernández , et al., “Meta‐Analysis of Genome‐Wide Association Studies for Cancer Therapy‐Related Cardiovascular Dysfunction and Functional Mapping Highlight an Intergenic Region Close to TP63,” Scientific Reports 14 (2024): 18413. 39117733 10.1038/s41598-024-69064-5 PMC11310459

[252] T. Lavabre‐Bertrand , L. Henry , S. Carillo , et al., “Plasma Proteasome Level Is a Potential Marker in Patients With Solid Tumors and Hemopoietic Malignancies,” Cancer 92 (2001): 2493–2500. 11745181 10.1002/1097-0142(20011115)92:10<2493::aid-cncr1599>3.0.co;2-f

[253] S. U. Sixt and B. Dahlmann , “Extracellular, Circulating Proteasomes and Ubiquitin—incidence and Relevance,” Biochimica Et Biophysica Acta 1782 (2008): 817–823. 18602990 10.1016/j.bbadis.2008.06.005

[254] D. Cardinale and M. T. Sandri , “Role of Biomarkers in Chemotherapy‐Induced Cardiotoxicity,” Progress in Cardiovascular Diseases 53 (2010): 121–129. 20728699 10.1016/j.pcad.2010.04.002

